# Resonance theory of vibrational polariton chemistry at the normal incidence

**DOI:** 10.1515/nanoph-2023-0685

**Published:** 2024-02-23

**Authors:** Wenxiang Ying, Michael A. D. Taylor, Pengfei Huo

**Affiliations:** Department of Chemistry, 6927 University of Rochester, 120 Trustee Road, Rochester, NY 14627, USA; Hajim School of Engineering, 6927 The Institute of Optics, University of Rochester, Rochester, NY 14627, USA

**Keywords:** vibrational strong coupling, polariton chemistry, normal incidence resonance

## Abstract

We present a theory that explains the resonance effect of the vibrational strong coupling (VSC) modified reaction rate constant at the normal incidence of a Fabry–Pérot (FP) cavity. This analytic theory is based on a mechanistic hypothesis that cavity modes promote the transition from the ground state to the vibrational excited state of the reactant, which is the rate-limiting step of the reaction. This mechanism for a single molecule coupled to a single-mode cavity has been confirmed by numerically exact simulations in our recent work in [J. Chem. Phys. 159, 084104 (2023)]. Using Fermi’s golden rule (FGR), we formulate this rate constant for many molecules coupled to many cavity modes inside a FP microcavity. The theory provides a possible explanation for the resonance condition of the observed VSC effect and a plausible explanation of why only at the normal incident angle there is the resonance effect, whereas, for an oblique incidence, there is no apparent VSC effect for the rate constant even though both cases generate Rabi splitting and forming polariton states. On the other hand, the current theory cannot explain the collective effect when a large number of molecules are collectively coupled to the cavity, and future work is required to build a complete microscopic theory to explain all observed phenomena in VSC.

## 1 Introduction

Recent experiments [1], [2], [3], [4], [5], [6] have demonstrated that chemical reaction rate constants can be suppressed [1], [2], [3], [4], [7], [8], [9] or enhanced [5], [6], [10] by resonantly coupling molecular vibrations to quantized radiation modes inside a Fabry–Pérot (FP) microcavity [11], [12], [13]. This effect has the potential to selectively slow down competing reactions [3] or speed up a target reaction, thus achieving mode selectivity and offering a paradigm shift in chemistry. Despite extensive theoretical efforts [8], [14], [15], [16], [17], [18], [19], [20], [21], [22], [23], [24], [25], [26], [27], [28], [29], [30], [31], [32], [33], [34], [35], [36], [37], [38], [39], [40], [41], the fundamental mechanism and theoretical understanding of the cavity-modified ground-state chemical kinetics remain elusive [14], [42], [43], [44]. To the best of our knowledge, there is no unified theory that can explain all of the observed phenomena in the vibrational strong coupling (VSC) experiments [14], including (1) The resonance effect, which happens when the cavity frequency matches the bond vibrational frequency, *ω*_c_ = *ω*_0_, but also only happens when the in-plane photon momentum is *k*_‖_ = 0 (the normal incidence), (2) The collective effect [1], [4], [5] which is the increase in the *magnitude* of VSC modification when increasing the number of molecules *N* (or concentration N/V ), (3) The driving by thermal fluctuations without optical pumping [1], [3]. (4) The isotropic disorder of the dipoles in the cavity, which is assumed in experiments with many molecules [14].

We aim to develop a microscopic theory to explain these observed VSC effects, especially focusing on understanding the resonance effect under normal incidence. Experimentally, only the resonance at normal incidence (*k*_‖_ = 0) gives rise to VSC effects on the rate constant, while a red-detuned cavity that has a light–matter resonance at *k*_‖_ > 0 (oblique incidence) does not give any VSC effect. This observation strongly suggests that forming Rabi splitting is not a *sufficient condition* for achieving the VSC-modified rate effect. Despite recent theoretical progress [18], [45], [46], the resonant condition under normal incidence remains *an unresolved question*.

In this work, we generalized our recently developed analytic Fermi’s golden rule (FGR) rate theory of VSC in Ref. [47] by incorporating many molecules and many cavity modes for both 1D FP cavities (with only 1D in the in-plane direction) and 2D FP cavities (with 2D in the in-plane direction) cases. In particular, we evaluated the photonic mode density of states (DOS) inside a 1D FP cavity and found that it gives rise to a van-Hove-type singularity at *k*_‖_ = 0; for a 2D FP cavity, it is found that due to the cavity modes with *k*_‖_ > 0 propagating outside a given cavity mode extent area, the modified photon mode DOS still remains dominant around the bottom of the dispersion band where *k*_‖_ = 0, which are the keys to account for the normal incidence condition of the VSC-modified chemical reaction rate constant. The current theory provides a possible explanation of the resonance condition for the observed VSC effect and provides a microscopic understanding of why only at the normal incident angle there is a resonance effect.

## 2 Model system

**Figure 1:**
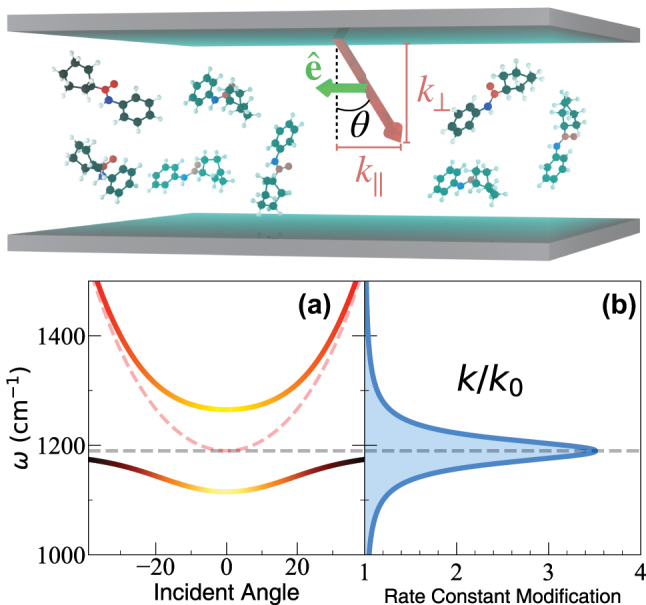
Top: Schematic illustration of the normal incidence condition for VSC-modified reactions. Bottom: (a) Schematic illustration of the dispersion relations of the cavity (red dashed line), the vibrational energy (gray dashed line), and hybrid polariton states (solid lines). (b) Schematic plot of reaction rate modification as a function of the cavity frequency *ω*_c_.

Let us consider *N* identical molecules coupled to many radiation modes inside a FP cavity,

(1) $$\begin{array}{ll} \hat{H} & =\overset{N}{\underset{j=1}{\sum }}\frac{{\hat{P}}_{j}^{2}}{2M}+V\left({\hat{R}}_{j}\right)+{\hat{H}}_{\nu }+{\hat{H}}_{\text{loss}}\left({\hat{q}}_{k},{\hat{x}}_{k,\zeta }\right)\\ & \;+\underset{k}{\sum }\frac{{\hat{p}}_{k}^{2}}{2}+\frac{\omega_{k}^{2}}{2}{\left({\hat{q}}_{k}+\frac{\lambda_{\text{c}}}{\omega_{k}}\cdot \overset{N}{\underset{j=1}{\sum }}\hat{\mu }\left({\hat{R}}_{j}\right)\cdot {\hat{e}}_{k}\right)}^{2}, \end{array}$$

where ${\hat{R}}_{j}$ is the reaction coordinate for the *j*th molecule, $V\left({\hat{R}}_{j}\right)$ is the ground state potential for each reaction molecule (a double well potential for this paper as is typical for VSC simulations [22], [27], [34], [39]), and $\hat{\mu }\left({\hat{R}}_{j}\right)$ is the dipole operator associated with the ground electronic state of reaction coordinate ${\hat{R}}_{j}$ (electronic permanent dipole). In particular, *φ*_*j*,**k**_ is the angle between $\hat{\mu }\left({\hat{R}}_{j}\right)$ and the field polarization direction ${\hat{e}}_{k}$ , where we only consider transverse electric (TE) polarization, such that $\hat{\mu }\left({\hat{R}}_{j}\right)\cdot {\hat{e}}_{k}=\mu \left({\hat{R}}_{j}\right)\cos\varphi_{j,k}$ . A schematic illustration is provided in the top panel of Figure 1.

The photonic wavevector **k** (also referred to as the field propagation direction) has two components, one perpendicular to the cavity mirror *k*_⊥_, and the other coplanar with the cavity *k*_‖_. The FP cavity has the following dispersion relation,

(2) $$\omega_{k}\left(k_{\|}\right)=\frac{c}{n_{\text{c}}}\sqrt{k_{⊥}^{2}+k_{\|}^{2}}=\frac{ck_{⊥}}{n_{\text{c}}}\sqrt{1+\tan^{2}\;\theta },$$

where *c* is the speed of light in vacuum, *n*_c_ is the refractive index of the cavity, *c*/*n*_c_ is the speed of the light inside the cavity, and *θ* is commonly referred to as the incident angle (where tan*θ* = *k*_‖_/*k*_⊥_), which is the angle of the photonic mode wavevector **k** relative to the norm direction of the mirrors (see the top panel of Figure 1 for a schematic illustration). In most of the VSC experiments, *n*_c_ ≈ 1.5 for the solution used inside the microcavity. Because *n*_c_ ≈ 1, it will not influence the order of the magnitude of our discussion. For simplicity, we explicitly drop *n*_c_ throughout this paper. Later, whenever we write *c* in an expression we should replace it with *c*/*n*_c_, in principle. When *k*_‖_ = 0 (or *θ* = 0), the photon frequency is

(3) $$\omega_{\text{c}}\equiv \omega_{k}\left(k_{\|}=0\right)=ck_{⊥}.$$

The cavity frequency *ω*_**k**_ in Eq. (1) is associated with the wavevector **k**, according to Eq. (2). Furthermore, ${\hat{q}}_{k}=\sqrt{\hbar /\left(2\omega_{k}\right)}\left({\hat{a}}_{k}^{†}+{\hat{a}}_{k}\right)$ and ${\hat{p}}_{k}=i\sqrt{\hbar \omega_{k}/2}\left({\hat{a}}_{k}^{†}-{\hat{a}}_{k}\right)$ , ${\hat{a}}_{k}$ and ${\hat{a}}_{k}^{†}$ are the photonic field annihilation and creation operators for mode **k**, respectively. The light–matter coupling strength is

(4) $$\lambda_{\text{c}}=\sqrt{1/\left(\epsilon_{0}V\right)},$$

where *ϵ*_0_ is the effective permittivity inside the cavity and V is the cavity quantization volume. Each reaction coordinate ${\hat{R}}_{j}$ is coupled to its own local phonon bath described by ${\hat{H}}_{\nu }$ . Each cavity mode ${\hat{q}}_{k}$ couples to its independent bath $\left\{{\hat{x}}_{k,\zeta }\right\}$ , accounting for the cavity loss. On the other hand, ${\hat{q}}_{k}$ also couples to the dipole of each molecule $\hat{\mu }\left({\hat{R}}_{j}\right)$ with a relative angle *φ*_*j*,**k**_. For 2D-FP cavities, we further define the angle between the dipole and the *k*_‖_ plane as *φ*_*j*_ which varies from 0 to *π*, and the angle between the field polarization and the projection of the dipole on the *k*_‖_ plane as *ϕ*_*j*,**k**_ which varies from 0 to 2*π*. It is easy to prove that cos *φ*_*j*,**k**_ = cos *φ*_*j*_ ⋅ cos *ϕ*_*j*,**k**_. For 1D FP cavity, *φ*_*j*,**k**_ will reduce to *φ*_*j*_ since *ϕ*_*j*,**k**_ = 0. Details of the Hamiltonian and a schematic illustration of the orientations of the dipole operator and field polarization vector are provided in the Supplementary Material, Section I.

Figure 1(a) presents a schematic illustration of the cavity dispersion relation in Eq. (2) (red dashed line). The molecular excitation dispersion (black dashed line) is insensitive to the incident angle and is a straight line, with energy *ℏω*_0_ (see Eq. (7)). These two dispersions hybridize due to the light–matter interactions, generating polariton dispersions (the upper and lower branches with solid curves) with the color coding indicating the character of the states, with purely photonic (red), purely vibrational (black), and hybridized (yellow to orange). Figure 1(b) presents a schematic illustration of the typical cavity detuning dependence of the rate constant modifications, with the highest intensity of the modification arising at the frequency when *ω*_c_ = *ω*_0_ (resonance condition at the normal incidence).

**Figure 2:**
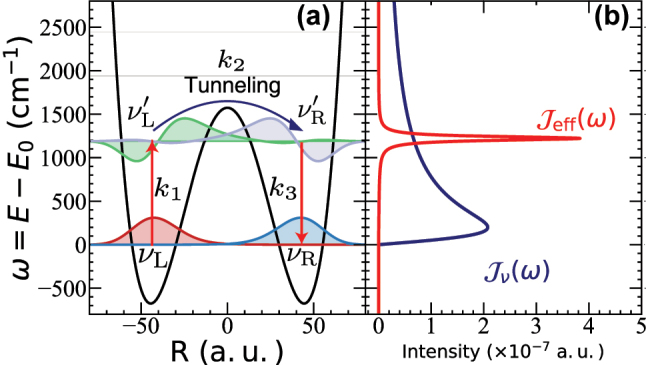
Potential energy surface for the reaction model. The red arrows represent the thermal activation process from the vibrational ground state, |*ν*_L_⟩, to the vibrationally excited state, $|\nu_{\text{L}}^{\prime }〉$ in the reactant well (left side of the barrier). Through the coupling between $|\nu_{\text{L}}^{\prime }〉$ and $|\nu_{\text{R}}^{\prime }〉$ , a chemical reaction occurs. Finally, the vibrational excited state $|\nu_{\text{R}}^{\prime }〉$ relaxed to the ground state |*ν*_R_⟩. (b) The effective spectral density *J*_eff_(*ω*) (red curve), corresponds to the cavity and its associated loss, compared to the phonon spectral density *J*_*ν*_(*ω*) (blue).

In this paper, we consider a reaction using a thermal barrier crossing model. Figure 2(a) presents the first few vibrational states of the double well model, where |*ν*_L_⟩ denotes the vibrational ground state of the reactant (left well), $|\nu_{\text{L}}^{\prime }〉$ denotes the vibrationally excited state of the reactant, and similar for the product (right well). The red arrow represents the thermal activation process from the vibrational ground state, |*ν*_L_⟩, to the vibrationally excited state, $|\nu_{\text{L}}^{\prime }〉$ in the reactant well. Then, through the coupling between $|\nu_{\text{L}}^{\prime }〉$ and $|\nu_{\text{R}}^{\prime }〉$ , a chemical reaction occurs. Finally, the vibrational excited state $|\nu_{\text{R}}^{\prime }〉$ relaxes to the ground state of the product |*ν*_R_⟩. The presence of the cavity mode ${\hat{q}}_{k}$ explicitly enhances the transition $|\nu_{\text{L}}〉\to |\nu_{\text{L}}^{\prime }〉$ . The symmetric double-well model [39] is used to model the reaction, with details in Supplementary Material, Section II. Figure 2(b) shows the phonon spectral density *J*_*ν*_(*ω*) (blue) adapted from Ref. [39] as well as the effective spectral density *J*_eff_(*ω*) (red) of the cavity and its associated environment that accounts for loss. Note that *J*_eff_(*ω*) resembles the Brownian oscillator spectral density that centers at a particular frequency. When its peak frequency is in resonance with the quantum vibrational frequency *ω*_0_, *J*_eff_(*ω*) could potentially accelerate the state-to-state quantum transitions $|\nu_{\text{L}}〉\to |\nu_{\text{L}}^{\prime }〉$ (as indicated by the red arrows in Figure 2(a)). Note that the actual experimental system might not be able to be modeled as a simple symmetric double well potential as shown in Figure 2. Nevertheless, we do expect the mechanism obtained from investigating this simple model to be insightful and characteristic of the VSC problems. Recent quantum dynamics simulations [39] using models with asymmetrical double well potential, a much higher reaction barrier than *ℏω*_01_, or coupling cavity to the spectator mode (which in turn couple to the reaction coordinate) do show a sharp resonance modification of the rate constant. We anticipate that the current mechanistic explanation can also be used to explain these sharp resonance features.

Consider a simplified reaction mechanism outside the cavity as $|\nu_{\text{L}}〉\overset{k_{1}}{\to }|\nu_{\text{L}}^{\prime }〉\overset{k_{2}}{\to }|\nu_{\text{R}}^{\prime }〉\overset{k_{3}}{\to }|\nu_{\text{R}}〉$ . Note that this is the quantum description of the reaction based on quantized states, whereas the classical description is a barrier crossing along the reaction coordinate. These vibrational diabatic states can be directly obtained by computing the eigenspectum of $V\left(\hat{R}\right)$ and then diabatizing it. The dominant pathway enhanced by VSC effects is through the first excited states [47]. The simplified mechanism for this reaction is that the thermal activation process causes the transition of $|\nu_{\text{L}}〉\to |\nu_{\text{L}}^{\prime }〉$ . Then the reaction occurs through the diabatic couplings between $|\nu_{\text{L}}^{\prime }〉$ and $|\nu_{\text{R}}^{\prime }〉$ , followed by a vibrational relaxation of the product state, $|\nu_{\text{R}}^{\prime }〉\to |\nu_{\text{R}}〉$ . The rate-limiting step for the entire process is *k*_1_, where *k*_2_ ≫ *k*_1_ such that the populations of both $|\nu_{\text{L}}^{\prime }〉$ and $|\nu_{\text{R}}^{\prime }〉$ reach a steady state (plateau in time), and from the steady-state approximation, the overall rate constant for the reaction is *k*_0_ ≈ *k*_1_. This steady-state behavior of the $|\nu_{\text{L}}^{\prime }〉$ and $|\nu_{\text{R}}^{\prime }〉$ states has recently been verified by numerically exact quantum dynamics simulations [47].

Considering many molecules, we focus on the single excitation subspace. This includes the ground state |*G*⟩ and *N* singly excited states |*ν*_*j*_⟩ (where *j* ∈ [1, *N*] labels the molecules), defined as

(5a) $$|G〉\equiv |\nu_{\text{L}}^{1}〉\ldots ⊗\ldots |\nu_{\text{L}}^{j}〉⊗\ldots |\nu_{\text{L}}^{N}〉,$$

(5b) $$|\nu_{j}〉\equiv |\nu_{\text{L}}^{1}〉\ldots ⊗\ldots |\nu_{\text{L}}^{\prime j}〉⊗\ldots |\nu_{\text{L}}^{N}〉.$$

The vibrational transition dipole matrix element is

(6) $$\mu_{\text{L}L^{\prime }}=〈\nu_{\text{L}}^{\prime j}|\mu \left({\hat{R}}_{j}\right)|\nu_{\text{L}}^{j}〉,$$

which is identical for all molecules *j*. When measuring the absorption spectra of the molecule, the optical response shows a peak at the quantum vibrational frequency

(7) $$\omega_{0}=\left(E_{L^{\prime }}-E_{\text{L}}\right)/\hbar .$$

In the singly excited manifold, the light–matter coupling term, $\propto \sum_{k,j}{\hat{q}}_{k}⊗\hat{\mu }\left({\hat{R}}_{j}\right)\cdot {\hat{e}}_{k}$ in Eq. (1), will hybridize the bright excitonic state and the photon-dressed ground states, generating the polariton states [48], [49]. When all dipoles are fully aligned with a given field polarization direction ${\hat{e}}_{k}$ , such that $\hat{\mu }\left({\hat{R}}_{j}\right)\cdot {\hat{e}}_{k}=\mu \left({\hat{R}}_{j}\right)$ , and under the resonance condition *ω*_**k**_(*k*_‖_) = *ω*_0_ for this particular **k**, the light–matter hybridization generates the upper and lower polariton states, causing the Rabi splitting expressed as

(8) $$\Omega_{\text{R}}=\sqrt{\frac{2\omega_{k}}{\hbar \epsilon_{0}}}\sqrt{\frac{N}{V}}\mu_{LL^{\prime }}\equiv 2\sqrt{N}g_{\text{c}}\cdot \sqrt{\omega_{k}},$$

where $g_{\text{c}}=\mu_{LL^{\prime }}\sqrt{1/\left(2\hbar \epsilon_{0}V\right)}$ is the Jaynes–Cummings [50] type coupling strength (without the $\sqrt{\omega_{k}}$ -dependence). Details of this standard analysis are provided in the Supplementary Material, Section III.

The formation of Rabi splitting/polariton states comes from a collective phenomenon, resulting in the well-known dependence of $\sqrt{N}$ or equivalently $\sqrt{N/V}$ for Ω_R_, which has been experimentally confirmed [4]. It has been estimated that there are *N* ∼ 10^6^ − 10^12^ molecules effectively coupled to the cavity mode [14], [16], [52] for recent VSC experiments [1], [4], and Ω_R_ ∼ 100 cm^−1^ when *ω*_0_ ≈ 1000 cm^−1^ for typical VSC experiments [4], [5]. Despite encouraging progress, what remains largely a mystery is how the collective light–matter coupling can induce the VSC modified rate constant [14]. Another less investigated area [18], [45], [46] is why forming polaritons at a finite incident angle does not necessarily lead to the change of the VSC kinetics, and the only observed VSC effects occur at *k*_‖_ = 0 (or *θ* = 0). This strongly hints that forming polariton states is not a sufficient condition for VSC-modified effects, and polariton states might not be the best representation for explaining the VSC modification, because the polariton states are present in both normal and oblique incidences, and yet only the former case result in the VSC modified rate constant.

## 3 Theoretical results

### 3.1 Analytic rate constant expression

To provide a microscopic mechanism of VSC-modified reactions, we *hypothesize* that the cavity modes enhance the transition from ground states to a vibrationally excited state manifold of the reactant, leading to an enhancement of the *steady-state population* of both the delocalized states on the reactant side and the excited states manifold on the product side (right well) $\left\{|\nu_{\text{R}}^{\prime j}〉\right\}$ , which then relax to the vibrational ground state manifold on the product side (right well), $\left\{|\nu_{\text{R}}^{j}〉\right\}$ . For a single molecule strongly coupled to a single cavity mode, our numerical simulations [47] have confirmed the validity of this hypothesis. The proposed reaction mechanism is represented below

(9) $$|G〉\overset{k_{1}}{\to }\left\{|\nu_{\text{L}}^{\prime j}〉\right\}\overset{k_{2}}{\to }\left\{|\nu_{\text{R}}^{\prime j}〉\right\}\overset{k_{3}}{\to }\left\{|\nu_{\text{R}}^{j}〉\right\},$$

among which *k*_1_ ≪ *k*_2_, *k*_3_. Note that in the current work, we only consider the single excitation subspace (where one particular vibration is excited). In real experiments, many molecules could be simultaneously excited [13], with a number $n_{\text{ex}}\approx N{\text{e}}^{-\beta \hbar \omega_{0}}$ , such that 1 ≪ *n*_ex_ ≪ *N*. For example, when *N* = 10^12^, *βℏω*_0_ ≈ 5, *n*_ex_ ∼ 10^9^. Future development is needed to fully account for such statistical distributions among molecules.

When the molecular system is originally in the Kramers low friction regime (before the Kramers turnover [53], [54], or the so-called energy diffusion-limited regime), the cavity enhancement of the rate constant *k*_1_ will occur [30], [31], [32], [34], [35], [39]. This has been extensively discussed in recent theoretical work [39], [47]. If we explicitly assume that *k*_1_ ≪ *k*_2_, *k*_3_, then $|G〉\overset{k_{1}}{\to }\left\{|\nu_{\text{L}}^{\prime j}〉\right\}$ is the *rate limiting step*, and the population of intermediate states will reach a steady-state behavior. As such, due to the steady-state approximation, the overall rate constant is [47]

(10) $$k\approx k_{1}=k_{0}+k_{\text{VSC}}\ll k_{2},k_{3},$$

where *k*_0_ is the chemical reaction rate constant outside the cavity, and *k*_VSC_ accounts for the pure cavity-induced effect. As this is a thermally activated reaction, there already exist some excited-state populations and transitions outside the cavity, which *k*_0_ accounts for. Note that Eq. (10) assumes that the pure cavity effect *k*_VSC_ can be added with *k*_0_, which is a *fundamental assumption* in the current theory.

To quantitatively express *k*_VSC_, we analyze the overall effect of the cavity and the photon loss environment on molecular systems by performing a normal mode transformation [55], [56], [57] to the Hamiltonian in Eq. (1) and obtaining an effective Hamiltonian, where now the cavity modes $\left\{{\hat{q}}_{k}\right\}$ and the photon loss bath modes $\left\{{\hat{x}}_{k,\zeta }\right\}$ (described by ${\hat{H}}_{\text{loss}}$ ) are transformed into effective photonic normal mode coordinates $\left\{{\hat{\tilde{x}}}_{k,\zeta }\right\}$ , which are collectively coupled to the system DOF through the following term,

(11) $${\hat{H}}_{\text{LM}}=\hat{S}⊗\underset{k}{\sum }{\hat{F}}_{k},$$

where $\hat{S}\equiv \sum_{j=1}^{N}\mu \left({\hat{R}}_{j}\right)\cdot \cos\varphi_{j}$ is the collective system operator which does not depend on **k**, as we used the relation cos *φ*_*j*,**k**_ = cos *φ*_*j*_ ⋅ cos *ϕ*_*j*,**k**_ to separate the **k**-dependent and **k**-independent components. For simplicity, we have assumed that *ϕ*_*j*,**k**_ → *ϕ*_**k**_ is *j*-independent for the 2D cavity case, which means the molecular dipoles are distributed in a 2D plane that perpendicular to the mirrors (which is naturally true for the 1D cavity case). Further, ${\hat{F}}_{k}=\cos\phi_{k}\cdot \sum_{\zeta }{\tilde{c}}_{k,\zeta }{\hat{\tilde{x}}}_{k,\zeta }$ is the stochastic force exerted by the **k**-th effective bath, $\left\{{\hat{\tilde{x}}}_{k,\zeta }\right\}$ are the normal modes of $\left\{{\hat{q}}_{k},{\hat{x}}_{k,\zeta }\right\}$ , and the coupling constants ${\tilde{c}}_{k,\zeta }$ as well as bath frequencies ${\tilde{\omega }}_{k,\zeta }$ are characterized by an effective spectral density,

(12) $$J_{\text{eff}}\left(\omega_{k},\omega \right)=\frac{\lambda_{\text{c}}^{2}\cos^{2}\phi_{k}\omega_{k}^{2}\tau_{\text{c}}^{-1}\omega }{{\left(\omega_{k}^{2}-\omega^{2}\right)}^{2}+\tau_{\text{c}}^{-2}\omega^{2}},$$

where *τ*_c_ is the cavity lifetime. Detailed derivation is provided in Supplementary Material, Section IV.

The rate constant change *k*_VSC_ in Eq. (10) originates from a purely cavity-induced effect, which promotes the transition from |*G*⟩ to the singly excited states manifold {|*ν*_*j*_⟩}. Note that this transition is mediated by the cavity operators ${\hat{F}}_{k}$ through the collective coupling between all molecules and the cavity modes, as is suggested by the light–matter coupling term in Eq. (11). We use FGR to estimate this transition rate constant. The coupling for this quantum transition is provided by $\hat{S}$ , and the transition is mediated by the effective photon bath operators ${\hat{F}}_{k}$ with their spectral densities *J*_eff_(*ω*_**k**_, *ω*) in Eq. (12). Using FGR to estimate the transition with the frequency *ω* = *ω*_0_ (the $|G〉\overset{k_{1}}{\to }\left\{|\nu_{\text{L}}^{\prime j}〉\right\}$ transition), and assuming that the pathways are completely independent (*i.e.*, no interference between pathways), we have the following expression for the overall reaction rate constant,

(13) $$\begin{array}{ll} k_{\text{VSC}}^{\text{D}} & =\frac{1}{N}\frac{2}{\hbar }\overset{N}{\underset{j=1}{\sum }}{\left|〈\nu_{j}|\hat{S}|G〉\right|}^{2}\cdot \underset{k}{\sum }P_{k}\cdot J_{\text{eff}}\left(\omega_{k},\omega_{0}\right)\cdot n\left(\omega_{0}\right)\\ & =\frac{4}{N}g_{N}^{2}\cdot \underset{k}{\sum }P_{k}\cdot \frac{\cos^{2}\phi_{k}\omega_{k}^{2}\tau_{\text{c}}^{-1}\omega_{0}}{{\left(\omega_{k}^{2}-\omega_{0}^{2}\right)}^{2}+\tau_{\text{c}}^{-2}\omega_{0}^{2}}\cdot n\left(\omega_{0}\right), \end{array}$$

where *D* denotes the dimension of the in-plane direction in a FP cavity. The collective Jaynes-Cummings-type [50] coupling strength $g_{N}^{2}$ (without cavity frequency dependence) is defined as

(14) $$g_{N}^{2}\equiv g_{\text{c}}^{2}\overset{N}{\underset{j=1}{\sum }}\cos^{2}\varphi_{j},$$

and the 1/*N* factor accounts for the normalized rate constant per molecule. Furthermore,

(15) $$n\left(\omega_{0}\right)=1/\left({\text{e}}^{\beta \hbar \omega_{0}}-1\right)\approx {\text{e}}^{-\beta \hbar \omega_{0}}$$

is the Bose–Einstein distribution function, where *β* = 1/(*k*_B_ *T*) with *k*_B_ as the Boltzmann constant and *T* as the temperature. For the typical parameters in VSC experiments, *ω*_0_ ≈ 1200 cm^−1^ and room temperature 1/*β* = *k*_B_ *T* ≈ 200 cm^−1^, such that *βℏω*_0_ ≫ 1. Finally, $P_{k}$ represents the thermal weighting factor for accessing the cavity mode *ω*_**k**_, with

(16) $$P_{k}=\frac{{\text{e}}^{-\beta \hbar \omega_{k}}}{Z},$$

and Z is the partition function such that $\sum_{k}P_{k}=1$ . Note that the same thermal average over different modes is also used in a recent study of electron transfer rate theory in Ref. [61]. Detailed derivations are provided in Section V of the Supplementary Material.

Under the continuous *k*_‖_ limit, one can replace the sum in Eq. (13) with an integral as $\sum_{k}f\left(k\right)\to \int \frac{dk^{\text{D}}}{{\left(\Delta k_{\|}\right)}^{\text{D}}}\;f\left(k\right)$ , where Δ*k*_‖_ is the spacing of the in-plane wavevector *k*_‖_ (or the *k*-space lattice constant). See Section VI of the Supplementary Material for details. For 1D FP cavities, Eq. (13) becomes

(17) $$\begin{array}{ll} k_{\text{VSC}}^{1\text{D}} & =\frac{4}{N}g_{N}^{2}\int \frac{dk}{\Delta k_{\|}}\;P_{k}\cdot \frac{\omega_{k}^{2}\tau_{\text{c}}^{-1}\omega_{0}}{{\left(\omega_{k}^{2}-\omega_{0}^{2}\right)}^{2}+\tau_{\text{c}}^{-2}\omega_{0}^{2}}\cdot n\left(\omega_{0}\right)\\ & =\frac{4}{N}g_{N}^{2}\int d\omega \int \frac{dk}{\Delta k_{\|}}\;\delta \left(\omega -\omega_{k}\right)\\ & \;\times P\left(\omega \right)\cdot \frac{\omega^{2}\tau_{\text{c}}^{-1}\omega_{0}}{{\left(\omega^{2}-\omega_{0}^{2}\right)}^{2}+\tau_{\text{c}}^{-2}\omega_{0}^{2}}\cdot n\left(\omega_{0}\right)\\ & =\frac{4}{N}g_{N}^{2}\int d\omega \;g_{1\text{D}}\left(\omega \right)P\left(\omega \right)\\ & \;\cdot \frac{\omega^{2}\tau_{\text{c}}^{-1}\omega_{0}}{{\left(\omega^{2}-\omega_{0}^{2}\right)}^{2}+\tau_{\text{c}}^{-2}\omega_{0}^{2}}\cdot n\left(\omega_{0}\right), \end{array}$$

where $P\left(\omega \right)={\text{e}}^{-\beta \hbar \omega }/Z$ (cf. Eq. (14)), and we have explicitly used cos *ϕ*_**k**_ = 1, and the 1D DOS is defined as

(18) $$g_{1\text{D}}\left(\omega \right)=\int \frac{dk}{\Delta k_{\|}}\;\delta \left(\omega -\omega_{k}\right).$$

Note that when all molecules are aligned with the cavity field polarization direction, such that cos *φ*_*j*_ = 1, $g_{N}^{2}=Ng_{\text{c}}^{2}$ (cf. Eq. (8)). When the dipole orientations are fully isotropic, $\sum_{j=1}^{N}\cos^{2}\varphi_{j}=N〈\cos^{2}\varphi 〉=N/3$ .

For 2D FP cavities, similarly, one has (cf. Eq. (13))

(19) $$\begin{array}{ll} k_{\text{VSC}}^{2\text{D}} & =\frac{4}{N}g_{N}^{2}\underset{k}{\sum }P_{k}\cdot \frac{\cos^{2}\phi_{k}\omega_{k}^{2}\tau_{\text{c}}^{-1}\omega_{0}}{{\left(\omega_{k}^{2}-\omega_{0}^{2}\right)}^{2}+\tau_{\text{c}}^{-2}\omega_{0}^{2}}\cdot n\left(\omega_{0}\right)\\ & =\frac{4}{N}g_{N}^{2}\int d\omega \;g_{2\text{D}}^{\prime }\left(\omega \right)P\left(\omega \right)\\ & \;\cdot \frac{\omega^{2}\tau_{\text{c}}^{-1}\omega_{0}}{{\left(\omega^{2}-\omega_{0}^{2}\right)}^{2}+\tau_{\text{c}}^{-2}\omega_{0}^{2}}\cdot n\left(\omega_{0}\right), \end{array}$$

where $g_{N}^{2}$ is defined in Eq. (14), and the 2D DOS weighted by cos^2^ *ϕ*_**k**_ is defined as

(20) $$g_{2\text{D}}^{\prime }\left(\omega \right)=\int \frac{dk^{2}}{{\left(\Delta k_{\|}\right)}^{2}}\;\delta \left(\omega -\omega_{k}\right)\cdot \cos^{2}\phi_{k},$$

whereas the standard 2D-DOS is defined as

(21) $$g_{2\text{D}}\left(\omega \right)=\int \frac{dk^{2}}{{\left(\Delta k_{\|}\right)}^{2}}\;\delta \left(\omega -\omega_{k}\right).$$

Note that $g_{2\text{D}}^{\prime }\left(\omega \right)=g_{2\text{D}}\left(\omega \right)/2$ (see the proof in Section VI of the Supplementary Material). Since there is only a 1/2 factor difference between $g_{2\text{D}}^{\prime }\left(\omega \right)$ and *g*_2D_(*ω*), which does not influence the shape of the rate profiles, we will regard $g_{2\text{D}}^{\prime }\left(\omega \right)$ as *g*_2D_(*ω*) in the following discussions.

We further define the accumulated spectral function A(ω) as follows, (cf. Eqs. (17) and (19))

(22) $$A\left(\tilde{\omega }\right)\equiv \int d\omega \;g_{\text{D}}\left(\omega \right)P\left(\omega \right)\cdot \frac{\omega^{2}\tau_{\text{c}}^{-1}\tilde{\omega }}{{\left(\omega^{2}-{\tilde{\omega }}^{2}\right)}^{2}+\tau_{\text{c}}^{-2}{\tilde{\omega }}^{2}},$$

and $k_{\text{VSC}}^{\text{D}}$ in Eq. (13) can then be written as

(23) $$k_{\text{VSC}}^{\text{D}}=\frac{4}{N}g_{N}^{2}\cdot A\left(\omega_{0}\right)\cdot n\left(\omega_{0}\right).$$

### 3.2 The resonance effect at the normal incidence

Next, we work to provide an analytic expression of A(ω) for the 1D and 2D FP cavities, which is one of the *main theoretical results* of this work. The 1D and 2D DOS defined in Eqs. (18) and (21) can be evaluated using the dispersion relation in Eq. (2).

For the one-dimensional FP cavity [46], if we ignore the influence of cavity loss ( ${\hat{H}}_{\text{loss}}$ in Eq. (1)), one can show that the DOS for the photonic modes (*D* = 1) is expressed as

(24) $$g_{1D}\left(\omega \right)=\frac{2}{c\Delta k_{\|}}\cdot \frac{\omega }{\sqrt{\omega^{2}-\omega_{\text{c}}^{2}}}\cdot \Theta \left(\omega -\omega_{\text{c}}\right),$$

where Θ(*ω* − *ω*_c_) is the Heaviside step function. Details of the derivations are provided in the Supplementary Material, Section VI. The DOS, *g*_1D_(*ω*), in Eq. (24) has a singularity at *ω* = *ω*_c_, which is known as (the first type of) the van-Hove-type singularity [58]. Such a concentrated peak in *g*_1D_(*ω*) at *ω* = *ω*_c_ has been numerically observed in Figure 1 of Ref. [46]. We will turn to the case of including the effects of photon propagation in the in-plane direction later in this section.

By using Eq. (24), we have the spectral function $A\left(\omega_{0}\right)$ in Eq. (22) for 1D FP cavities as follows,

(25) $$\begin{array}{ll} A\left(\omega_{0}\right) & =\frac{1}{Z}\int d\omega \;g_{1\text{D}}\left(\omega \right)\cdot {\text{e}}^{-\beta \hbar \omega }\frac{\omega^{2}\tau_{\text{c}}^{-1}\omega_{0}}{{\left(\omega^{2}-\omega_{0}^{2}\right)}^{2}+\tau_{\text{c}}^{-2}\omega_{0}^{2}}\\ & =\frac{2}{c\Delta k_{\|}Z}\overset{\omega_{m}}{\underset{\omega_{\text{c}}}{\int }}d\omega \;\frac{\omega \cdot {\text{e}}^{-\beta \hbar \omega }}{\sqrt{\omega^{2}-\omega_{\text{c}}^{2}}}\frac{\omega^{2}\tau_{\text{c}}^{-1}\omega_{0}}{{\left(\omega^{2}-\omega_{0}^{2}\right)}^{2}+\tau_{\text{c}}^{-2}\omega_{0}^{2}}, \end{array}$$

where *ω*_*m*_ → ∞ is the cutoff frequency. The integral in Eq. (25) gives a finite value despite the singularity in *g*_1D_(*ω*), because only the contribution from *ω* = *ω*_c_ survives. At the same time, $Z=\sum_{k}{\text{e}}^{-\beta \hbar \omega_{k}}=\int d\omega \;g_{1\text{D}}\left(\omega \right){\text{e}}^{-\beta \hbar \omega }\approx 2{\text{e}}^{-\beta \hbar \omega_{\text{c}}}/\left(c\Delta k_{\|}\right)$ , so 1/Z cancels the ${\text{e}}^{-\beta \hbar \omega_{\text{c}}}$ and the 2/(*c*Δ*k*_‖_) factor that arises from the integral. This leads to an approximate analytic expression of $A\left(\omega_{0}\right)$ for 1D FP cavity case as follows

(26) $$A\left(\omega_{0}\right)\approx \frac{\omega_{\text{c}}^{2}\tau_{\text{c}}^{-1}\omega_{0}}{{\left(\omega_{\text{c}}^{2}-\omega_{0}^{2}\right)}^{2}+\tau_{\text{c}}^{-2}\omega_{0}^{2}}.$$

We have also numerically evaluated Eq. (25) and compared it with Eq. (26) for the VSC rates, presented in Figure S2 of the Supplementary Material, which shows a nearly identical behavior. The above theoretical results also suggest that for a 1D cavity, the commonly used single mode approximation [22], [27], [39] is indeed valid, because only the mode of frequency *ω*_c_ survives. Using the expression of $A\left(\omega_{0}\right)$ (Eq. (26)) in the rate constant expression of Eq. (23) and taking the limit of *N* = 1, one obtains the previous result of *k*_VSC_ (see Eq. (35)) for a single molecule coupled to a single mode in Ref. [47]. We should remind the reader that all of the existing VSC experiments were conducted with 2D cavities.

**Figure 3:**
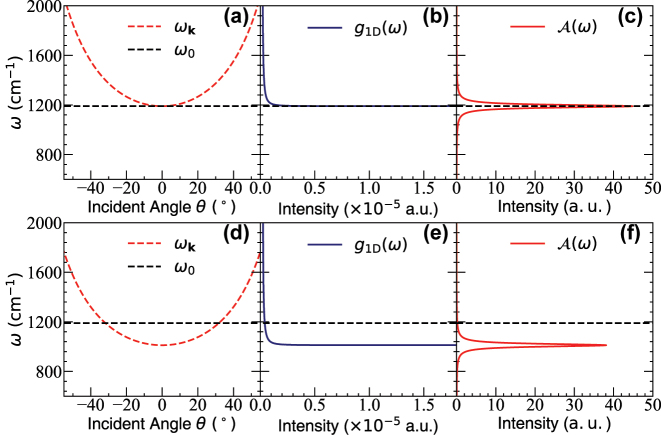
Dispersion relation, DOS, and the accumulated spectral function A(ω) for a 1D cavity. (a) The cavity dispersion relation (Eq. (2)), (b) the schematic 1D DOS *g*_1D_(*ω*) (Eq. (24)) and (c) the 1D accumulated spectral function A(ω) (Eq. (22), evaluated using Eq. (25)) for the normal incidence case *ω*_c_ = *ω*_0_, where the resonance condition is reached at *θ* = 0. (d)–(f) corresponds to the red-detuned case (oblique incidence), with *ω*_c_ = 0.85 *ω*_0_, whose resonance condition is reached at *θ* ≈ 32°. The cavity lifetime is taken as *τ*_c_ = 200 fs.

Figure 3 presents the cavity dispersion relation of *ω*_**k**_(*θ*) (see Eq. (2)) in panels (a) and (d), the 1D DOS *g*_1D_(*ω*) (see Eq. (24)) in panels (b) and (e), and the 1D accumulated spectral function A(ω) (see Eq. (22)) which is directly proportional to *k*_VSC_ in panels (c) and (f). In panels (a)–(c), one can clearly see that under the normal incident condition *ω*_**k**_ = *ω*_0_ at *θ* = 0, A(ω) is maximized at *ω*_c_ = *ω*_0_ and accordingly, the rate constant will also maximize based on the FGR expression (Eq. (13)). In the detuned case (*ω*_c_ ≠ *ω*_0_ or |*θ*| > 0) in panels (d)–(f), the intensity of A(ω) still peaks at *θ* = 0, but the value of $A\left(\omega_{0}\right)$ diminishes at the “resonance condition” *ω*_**k**_ = *ω*_0_ (for generating Rabi splitting).

This analysis also provides a possible explanation for the resonance effect at normal incidence (*k*_‖_ = 0) for a 1D FP cavity. In Eq. (26), it is clear that the peak of this function is located at *ω*_c_ = *ω*_0_ for *k*_‖_ = 0. Thus, the VSC-modified rate constant occurs only when *ω*_c_ = *ω*_0_. This is because there is a van-Hove-type singularity [58] in the 1D DOS, *g*_1D_(*ω*), which manifests itself as the $1/\sqrt{\omega^{2}-\omega_{\text{c}}^{2}}$ term in Eq. (25), such that the integral only survives and gives a finite value at *ω* = *ω*_c_, and at *ω* > *ω*_c_, the integral becomes vanishingly small.

However, directly extending this simple consideration for the DOS cannot explain the normal incidence condition for a 2D FP cavity (even when only considering the TE polarization direction). This is because the 2D DOS *g*_2D_(*ω*) does not have any singularity. Specifically, the DOS for the photonic modes inside a 2D FP cavity is expressed as

(27) $$g_{2\text{D}}\left(\omega \right)=\frac{2\pi }{{\left(c\Delta k_{\|}\right)}^{2}}\cdot \omega \cdot \Theta \left(\omega -\omega_{\text{c}}\right),$$

where Section VI of the Supplementary Material contain more details of this derivation.

For the 2D cavity case, one needs to consider beyond the simple DOS argument. Note that the photon loss associated with the lifetime *τ*_c_ only considers the loss in the *k*_⊥_ direction. What we have not explicitly considered before was the photon traveling outside a mode area along the *k*_‖_ direction. Let D be the effective lateral size of a given mode (which is not the cavity length), and L be the mirror distance (along the *k*_⊥_ direction in Figure 1), so the effective quantization volume (per mode) $V=L\cdot D^{2}$ . The mode lifetime can be estimated as

(28) $$\tau_{\|}\left(k_{\|}\right)=\frac{D}{c\cdot \sin\theta }=\frac{D\sqrt{k_{⊥}^{2}+k_{\|}^{2}}}{c\cdot k_{\|}}=\frac{D}{c^{2}}\cdot \frac{\omega_{k}}{k_{\|}},$$

which is propotional to $k_{\|}^{-1}$ when *k*_‖_ ≪ *k*_⊥_, and the associated rate constant is $\Gamma_{10}^{\prime }=1/\tau_{\|}$ (which corresponding to the photon loss of |1_**k**_⟩ → |0_**k**_⟩). Note that *τ*_‖_ differs from the cavity lifetime *τ*_c_ introduced previously. Specifically, *τ*_‖_ accounts for thermal photon traveling outside a coupling area associated with a given mode *ω*_**k**_ in the in-plane direction, *τ*_c_ describes the loss channel *only* due to the escaping of the photon with a direction that is perpendicular to the mirror surface *k*_⊥_ (and was introduced through the ${\hat{H}}_{\text{loss}}$ term, which was assumed to be identical for all cavity modes *ω*_**k**_, being independent of *k*_‖_).

An estimation for D is provided as follows. As mentioned before, the typical values for the VSC experiments are *N* ≈ 10^6^ ∼ 10^12^ [14], [16], [52], which is the effective number of molecules *per mode* (see estimations in Ref. [52]). The effective density is estimated to be $N/V\approx 10^{20}$ cm^−3^ [59]. Using $V=L\cdot D^{2}$ , and the typical value for the mirror distance L=1μ m, we have $D\approx 10^{-1}∼100\;\mu$ m (or 10^2^ ∼ 10^5^ nm), which agrees with the numerical simulation in a FP cavity based on eigenfrequency analysis of the scalar Helmholtz equation [51]. With the range of D , one can also estimate the range of D/c≈1∼100 fs. For example, when D∼300nm , $D/c∼10^{-15}\;s^{-1}=1\;fs$ . Note that D is different than the typical length of the cavity in the in-plane direction (which is on the order of mm [6], [8]). For a photon traveling outside a particular cavity mode area, it is still within the cavity quantization area that contains many modes. On the other hand, *τ*_c_ usually varies from 100 fs [3] to 5 ps [60] in typical VSC experiments.

Note that the term ${\text{e}}^{-\beta \hbar \omega_{k}}$ in Eq. (16) originates from the photon field thermal distribution, which can also be interpreted as the ratio between two photonic transition rate constants according to the detailed balance relation, *i.e.*,

(29) $${\text{e}}^{-\beta \hbar \omega_{k}}=\Gamma_{01}/\Gamma_{10},$$

where Γ_01_ is the rate for the |0_**k**_⟩ → |1_**k**_⟩ photonic Fock states transition due to thermal excitation, and Γ_10_ = 1/*τ*_c_ is the cavity loss rate along the *k*_⊥_ direction (associated with |1_**k**_⟩ → |0_**k**_⟩), which was assumed to be identical for all **k** modes. Note that all of the above-mentioned excitation and decay processes are related to the thermally activated radiation (thermal photon), and not related to the pumping with an external radiation field. To account for the additional effect of photon propagating outside a given area associated with a specific mode *ω*_**k**_, we modify the detailed balance relation (in Eq. (29)) by replacing the original $P_{k}$ with $P_{\text{eff}}\left(\omega_{k}\right)$ , defined as follows

(30) $$P_{\text{eff}}\left(\omega_{k}\right)=\frac{1}{Z_{\text{eff}}}\frac{\Gamma_{01}}{\Gamma_{10}+\Gamma_{10}^{\prime }}=\frac{1}{Z_{\text{eff}}}\frac{\tau_{\text{c}}^{-1}{\text{e}}^{-\beta \hbar \omega_{k}}}{\tau_{\text{c}}^{-1}+\tau_{\|}^{-1}},$$

where *τ*_‖_ (defined in Eq. (28)) is *k*_‖_-dependent. This can also be viewed as putting a $\tau_{\text{c}}^{-1}/\left(\tau_{\text{c}}^{-1}+\tau_{\|}^{-1}\right)$ correction factor to $P_{k}$ in Eq. (16), where *τ*_‖_ explicitly depends on *k*_‖_ (Eq. (28)). Further, the partition function is also modified as $Z\to Z_{\text{eff}}=\sum_{k}\tau_{\text{c}}^{-1}{\text{e}}^{-\beta \hbar \omega_{k}}/\left(\tau_{\text{c}}^{-1}+\tau_{\|}^{-1}\right)$ . As expected, when *k*_‖_ = 0, $\tau_{\|}^{-1}=0$ , one should have $P_{\text{eff}}\left(\omega_{k}\right)\to P_{k}={\text{e}}^{-\beta \hbar \omega_{k}}/Z$ . Note that in Eq. (30), we have not considered the effect of photon leaving from the mode **k**′ and re-entering into the mode **k**. This should be viewed as the limitation of the current theory. Future work is needed to consider this effect.

Using $P_{\text{eff}}\left(\omega_{k}\right)$ in Eq. (30), the accumulated spectral function $A\left(\omega_{0}\right)$ in Eq. (22) for 2D cavity is modified as

(31) $$\begin{array}{ll} A\left(\omega_{0}\right) & =\int d\omega \;g_{2\text{D}}\left(\omega \right)P_{\text{eff}}\left(\omega \right)\cdot \frac{\omega^{2}\tau_{\text{c}}^{-1}\omega_{0}}{{\left(\omega^{2}-\omega_{0}^{2}\right)}^{2}+\tau_{\text{c}}^{-2}\omega_{0}^{2}}\\ & =\frac{1}{Z_{\text{eff}}}\frac{2\pi }{{\left(c\Delta k_{\|}\right)}^{2}}\overset{\omega_{m}}{\underset{\omega_{\text{c}}}{\int }}d\omega \;F\left(\omega \right)\cdot \frac{\omega^{2}\tau_{\text{c}}^{-1}\omega_{0}}{{\left(\omega^{2}-\omega_{0}^{2}\right)}^{2}+\tau_{\text{c}}^{-2}\omega_{0}^{2}}, \end{array}$$

where we used $P_{\text{eff}}\left(\omega \right)$ in Eq. (31) and *τ*_‖_(*ω*) in Eq. (28), and we further define the following weighting factor

(32) $$F\left(\omega \right)\equiv \frac{\tau_{\text{c}}^{-1}\omega {\text{e}}^{-\beta \hbar \omega }}{\tau_{\text{c}}^{-1}+{\left[\tau_{\|}\left(\omega \right)\right]}^{-1}}.$$

This F(ω) takes a sharp maximum at *k*_‖_ = 0 and decays quickly when *k*_‖_ increases, because $\Gamma_{10}^{\prime }$ increases quickly as *k*_‖_ increases. This means that for a 2D cavity, as used in all existing VSC experiments, the VSC-modified rate constant is still maximized around *ω*_**k**_(*k*_‖_ = 0) = *ω*_c_ = *ω*_0_, fulfilling the normal incidence condition. Note that the correction factor $\tau_{\text{c}}^{-1}/\left(\tau_{\text{c}}^{-1}+\tau_{\|}^{-1}\right)$ can also be applied to the 1D FP cavity but does not introduce any difference in Figure 3, due to the van-Hove singularity in the DOS (see Eq. (24)) which dominates the entire integral, forcing $P_{\text{eff}}\left(\omega_{k}\right)\to P_{k}$ (as *τ*_‖_ → ∞ when *k*_‖_ = 0).

**Figure 4:**
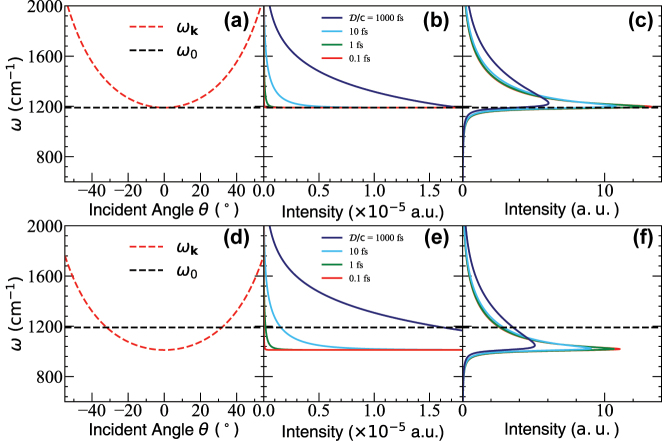
Same as Figure 3, but with a 2D FP cavity. (a) Same as Figure 3(a). (b) The weighting factor F(ω) (see Eq. (32)) under different D/c values, where D/c=0.1 fs corresponds to D≈0.03 µm, D/c=1000 fs corresponds to D≈300μ m. (c) The accumulated spectral function $A\left(\tilde{\omega }\right)$ (see Eq. (22)) for the normal incidence case *ω*_c_ = *ω*_0_, where the resonance condition is reached at *θ* = 0. (d)–(f) corresponds to the red-detuned case (oblique incidence), with *ω*_c_ = 0.85 *ω*_0_, whose resonance condition is reached at *θ* ≈ 32°. The cavity lifetime is taken as *τ*_c_ = 200 fs.

Figure 4 presents the cavity dispersion relation of *ω*_**k**_(*θ*) (see Eq. (2)) in panels (a) and (d), the weighting factor F(ω) (see Eq. (32)) in panels (b) and (e), and the 2D accumulated spectral function A(ω) (see Eq. (22)) for the 2D cavity case in panels (c) and (f). Figure 4(b) shows the numerical behavior of the weighting factor F(ω) under different D/c values (see Eq. (28)), among which D/c=1000 fs, 10 fs, 1 fs, and 0.1 fs, corresponding to $D=3\times 10^{5}$ nm, 3 × 10^3^ nm, 300 nm, and 30 nm, respectively. All are within the reasonable range of D values discussed previously. One can see that the maximal contribution still comes from *k*_‖_ = 0, although no singularity is present. Moreover, the width becomes narrower as D/c decreases. Note that c/D is usually a very large quantity, so that when the incident angle *θ* is slightly larger, $\Gamma_{10}^{\prime }\gg \Gamma_{10}$ becomes dominant.

Figure 4(c) presents the behavior of the accumulated spectral function A(ω) , which is calculated by evaluating Eq. (22) numerically using trapezoidal integration within the region of *ω*_c_ ≤ *ω*_**k**_ ≤ 5*ω*_c_ using 4 × 10^6^ grid points, where numerical convergence is carefully checked. One can see that A(ω) peaks at *ω* > *ω*_c_ when D/c is large, and gradually moves to *ω* = *ω*_c_ when D/c decreases and $\Gamma_{10}^{\prime }$ dominates the behavior for *k*_‖_ > 0. Additionally, compared to Figure 3(c), here A(ω) tails towards the higher energy. This is because the weighting factor F(ω) is not truly singular at *ω*_c_. The smaller the D/c value, the sharper A(ω) will be. When taking the limit of D/c→0 , Figure 4(c) reduces back to Figure 3(c). On the other hand, when D/c→∞ , there will be no loss in the in-plane direction, corresponding to a much wider A(ω) (see the dark blue curve in Figure 4(c–f)), which is only bounded by e^−*βℏω*^. Under this condition, A(ω) still peaks at a particular frequency, but with *ω*_c_ > *ω*_0_. Figure 4(d–f) corresponds to the red-detuned case under oblique incidence, where *ω*_c_ = 0.85*ω*_0_.

With the above analysis, we have theoretically justified why the VSC-modified chemical kinetics only occurs at the normal incidence when *ω*_c_ = *ω*_0_ for a 2D FP cavity, which agrees with experimental observations [1], [11], [12], [13]. This is because even though there is no singularity in *g*_2D_(*ω*), the photons propagating outside the mode area along the *k*_‖_ direction force the 2D cavity spectra function A(ω) to peak at *ω* = *ω*_c_, forcing the normal incidence condition. The condition for observing Rabi splitting (see Eq. (8)), on the other hand, is $\omega_{k}=\omega_{\text{c}}\sqrt{1+\tan^{2}\theta }=\omega_{0}$ for any *θ* ≥ 0. Although the modes with *θ* > 0 barely contribute to *k*_VSC_, the mode density is *finite* (see Figure 3(e)) and for *ω*_0_ > *ω*_c_ there will *always* be a mode available that satisfies *ω*_**k**_ = *ω*_0_, generating Rabi splitting at *θ* > 0. As such, the theory provides a step forward towards understanding the fundamental difference between the condition for forming the Rabi splitting and that of the VSC resonance modification of the rate constant. This explains the experimentally observed resonance phenomena [11], [14] that occur only at *ω*_c_ = *ω*_0_ at the normal incident angle when *k*_‖_ = 0 (or *θ* = 0), but not at a finite angle of *θ* even though the resonance condition for generating Rabi splitting is fulfilled.

### 3.3 No apparent collective effect

For our discussion on collectivity, we begin by considering the FGR expression in Eq. (13). For simplicity, we just focus on the 1D cavity case, since for 2D cavity there is no apparent collective effect either. If all the molecules’ dipoles are perfectly aligned with the cavity field polarization direction, then cos*φ*_*j*_ = 1 for all molecules, *j*, and $\hat{S}=\sum_{j}\mu \left({\hat{R}}_{j}\right)$ . Evaluating Eq. (23) using Eqs. (25) and (26) leads to

(33) $$k_{\text{VSC}}^{1\text{D}}\approx \frac{1}{N}\cdot 4Ng_{\text{c}}^{2}\omega_{\text{c}}^{2}\cdot \frac{\tau_{\text{c}}^{-1}\omega_{0}}{{\left(\omega_{\text{c}}^{2}-\omega_{0}^{2}\right)}^{2}+\tau_{\text{c}}^{-2}\omega_{0}^{2}}\cdot {\text{e}}^{-\beta \hbar \omega_{0}},$$

where we have explicitly approximated $n\left(\omega_{0}\right)\approx {\text{e}}^{-\beta \hbar \omega_{0}}$ (cf. Eq. (16)). As a special case of Eq. (33), when *ω*_c_ = *ω*_0_, Eq. (33) becomes

(34) $$k_{\text{VSC}}^{1\text{D}}=\frac{1}{N}\Omega_{\text{R}}^{2}\cdot \tau_{\text{c}}\cdot {\text{e}}^{-\beta \hbar \omega_{0}},$$

where $\Omega_{\text{R}}=2\sqrt{N}g_{\text{c}}\cdot \sqrt{\omega_{0}}$ . The cavity quality factor is often defined as $Q=\tau_{\text{c}}^{-1}\omega_{0}$ for the resonance condition. For the recent VSC experiment by Ebbesen [3], the typical values for these parameters are *τ*_c_ ≈ 100 fs (reading from a width of $\Gamma_{\text{c}}=\tau_{\text{c}}^{-1}\approx 53$ cm^−1^ of the cavity transmission spectra). If the cavity frequency is *ω*_c_ = *ω*_0_ = 1200 cm^−1^, then the quality factor is *Q* ≈ 22.6.

However, for the current theory in Eq. (34), the overall rate constant would not explicitly depend on *N* (Eq. (33)), meaning that only for the small *N* and strong coupling between molecules and the cavity mode there will be an appreciation amount of the cavity-modified effect. This is in contrast to the experimental observation of the collective effect and should be viewed as a major limitation of current theory. This limitation could be related to the fact that we have only considered the case of single excitation subspace in our theory, whereas in the experiments, a total of $n_{\text{ex}}\approx N{\text{e}}^{-\beta \hbar \omega_{0}}$ molecules could be simultaneously excited [13] due to the thermal statistics. Future work is needed for considering multiple excitations in *n*_ex_ vibrations and the rate constant theory in this scenario.

When considering the disorder of the orientation between the dipole and the cavity field polarization direction, the FGR rate in Eq. (33) becomes

$$k_{\text{VSC}}^{1\text{D}}=4g_{\text{c}}^{2}〈\cos^{2}\varphi 〉\cdot \frac{\tau_{\text{c}}^{-1}\omega_{\text{c}}^{2}\omega_{0}}{{\left(\omega_{\text{c}}^{2}-\omega_{0}^{2}\right)}^{2}+\tau_{\text{c}}^{-2}\omega_{0}^{2}}\cdot {\text{e}}^{-\beta \hbar \omega_{0}},$$

upon statistical averaging of dipole orientations. For fully isotropically distributed dipoles, ⟨ cos^2^ *φ*⟩ = 1/3.

### 3.4 Resonance behavior of *k*_VSC_

We want to demonstrate the numerical behavior of the current theory predicted by Eqs. (25) and (31). Because the current theory lacks the collective effect, we take the *N* = 1 limit and scale up the coupling strength between a single molecule and the cavity modes, as most previous work does [22], [23], [39]. This leads to the expression of (cf. Eq. (33))

(35) $$k_{\text{VSC}}^{1\text{D}}=\Omega_{\text{R}}^{2}\cdot \frac{\omega_{\text{c}}\tau_{\text{c}}^{-1}\omega_{0}}{{\left(\omega_{\text{c}}^{2}-\omega_{0}^{2}\right)}^{2}+\tau_{\text{c}}^{-2}\omega_{0}^{2}}\cdot {\text{e}}^{-\beta \hbar \omega_{0}}$$

under the single mode limit (or under the 1D cavity case, see Eqs. (25) and (26)). When further considering the presence of homogeneous or inhomogeneous broadening of the molecular system, the FGR expression will be a convolution between the original FGR expression, which does not considering the broadening for the *ω*_0_ (for example, Eq. (33)), and a broadening function (assumed to be a Gaussian), expressed as follows [47]

(36) $$k_{\text{VSC}}^{1\text{D}}=\overset{\infty }{\underset{0}{\int }}d\omega \;\kappa^{1\text{D}}\left(\omega \right)G\left(\omega -\omega_{0}\right),$$

where

(37a) $$\kappa^{1\text{D}}\left(\omega \right)=\Omega_{\text{R}}^{2}\cdot \frac{\omega_{\text{c}}\tau_{\text{c}}^{-1}\omega }{{\left(\omega_{\text{c}}^{2}-\omega^{2}\right)}^{2}+\tau_{\text{c}}^{-2}\omega^{2}}\cdot {\text{e}}^{-\beta \hbar \omega },$$

(37b) $$G\left(\omega -\omega_{0}\right)=\frac{1}{\sqrt{2\pi \sigma^{2}}}\exp\left[-\frac{{\left(\omega -\omega_{0}\right)}^{2}}{2\sigma^{2}}\right],$$

where *σ* is the variance of the Gaussian.

As expected, the *k*_VSC_ expression in Eq. (33) should contain several characteristic physical constants, including the speed of light *c* in *ω*_c_ (see Eq. (3)) as it is related to light–matter interaction, Planck’s constant *ℏ* in *g*_c_ (see Eq. (8)) as it should be a quantum theory, and Boltzmann’s constant *k*_B_ in *n*(*ω*_0_) as it is a thermally activated theory. We adopt a model system used in Ref. [39] to demonstrate the basic trend of *k*_VSC_ predicted by the current theory. The schematic of the model is provided in Figure 2, whereas the details are provided in Supplementary Material, Section II.

To obtain the numerically exact rate constant for the same model, we use hierarchical equations of motion (HEOM) to simulate the population dynamics and obtain the VSC-modified rate constant, with the details provided in Section VII of the Supplementary Material. The HEOM simulation requires a linear system-bath coupling Hamiltonian. To this end, we follow the previous work [22], [39] and assume that the dipole operator is linear, $\mu \left(\hat{R}\right)=\hat{R}$ . As a result, the light–matter coupling term in Eq. (1) (for a single molecule case) is simplified as $\omega_{\text{c}}{\hat{q}}_{\text{c}}\lambda \cdot \mu \left(\hat{R}\right)=\omega_{\text{c}}\lambda {\hat{q}}_{\text{c}}\hat{R}$ . Further, we follow Ref. [39] by defining the normalized light–matter coupling strength as below,

(38) $$\eta_{\text{c}}=\sqrt{\frac{1}{2\hbar \omega_{\text{c}}}}\lambda =\frac{\Omega_{\text{R}}}{2\omega_{\text{c}}\mu_{LL^{\prime }}}.$$

We use a similar range of *η*_c_ as used in Ref. [39].

The forward rate constant from the HEOM simulation is obtained by evaluating [39], [47]

(39) $$k=-\underset{t\to t_{\text{p}}}{\lim}\frac{{\dot{P}}_{R}\left(t\right)}{P_{R}\left(t\right)+\chi_{\text{eq}}\cdot \left[P_{R}\left(t\right)-1\right]},$$

where $\chi_{\text{eq}}\equiv P_{R}/P_{P}$ denotes the ratio of equilibrium population between the reactant and product, see Section VII of the Supplementary Material. The time derivative ${\dot{P}}_{R}\left(t\right)$ in Eq. (39) is evaluated numerically. For the symmetric double potential model considered in this work, *χ*_eq_ = 1. The limit *t* → *t*_p_ represents that the dynamics have already entered the rate process regime (linear response regime) and *t*_p_ represents the “plateau time” of the time-dependent rate which is equivalent to a flux-side time correlation function formalism. One can also view Eq. (39) as the flux-side correlation function that provides the time-dependent rate constant *k*(*t*), which captures both the initial transient dynamics (the oscillatory behaviors of *k*(*t*)) and the longer time rate process (plateau of *k*(*t*_p_)). For the FGR-based theory (Eq. (35)), we use the value of the *k*_0_ (outside the cavity rate constant) obtained from the HEOM simulation and report *k*/*k*_0_ = 1 + *k*_VSC_/*k*_0_.

We report the numerical value of *k*/*k*_0_ as a function of the cavity frequency *ω*_c_. For the rate constant predicted by FGR, we only report the value of *k*/*k*_0_ = 1 + *k*_VSC_/*k*_0_ (see Eq. (10)), where *k*_VSC_ is evaluated using Eq. (36), and the variance defined in Eq. (37b) is estimated as *σ* = 30.74 cm^−1^ for the model parameters we used. See Supplementary Material, Section VII for details. And we directly use the numerical result of *k*_0_ obtained from the HEOM simulation.

**Figure 5:**
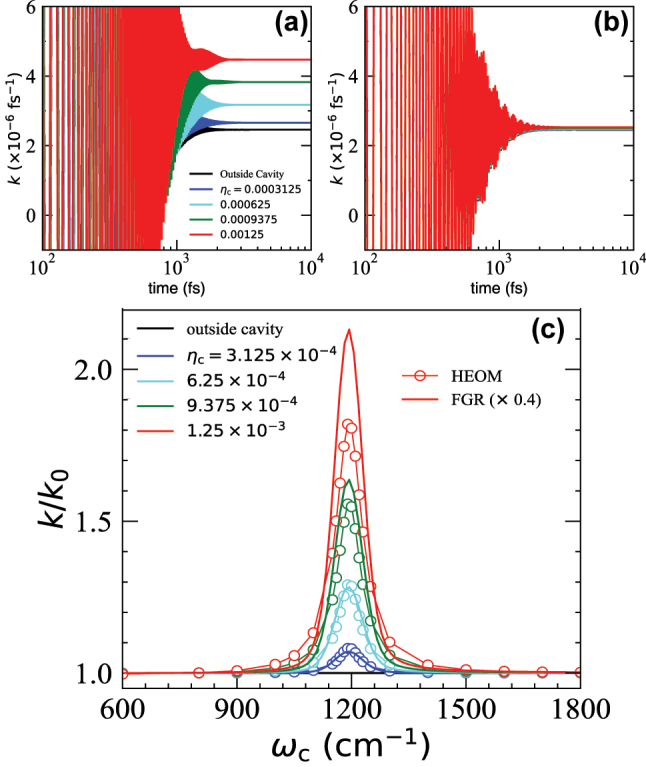
Numerically exact simulation and the analytic FGR results of the rate constant. (a) The flux-side correlation functions computed by HEOM at resonance (with *ω*_c_ = *ω*_0_ = 1190 cm^−1^). (b) The flux-side correlation functions are calculated by HEOM but off-resonance (with *ω*_c_ = 1000 cm^−1^). (c) The profile of the resonant VSC rate constant *k*/*k*_0_ as a function of *ω*_c_ with different light–matter coupling strengths, *η*_c_, obtained by FGR expressed in Eq. (36) (solid lines) and HEOM simulations (open circles with guiding thin lines), respectively. The cavity lifetime is set to be *τ*_c_ = 200 fs.

Figure 5 presents the numerical simulations of the rate constant from HEOM as well as the FGR results. Figure 5(a) presents *k*(*t*) for the resonant case when *ω*_c_ = *ω*_0_, at various light–matter coupling strengths *η*_c_. One can see the plateau value of *k*(*t*) increases as *η*_c_ increases. Figure 5(b) presents the case where *ω*_c_ < *ω*_0_ where *ω*_c_ = 1000 cm^−1^, and there is no apparent *η*_c_ dependence of *k*(*t*), indicating that the coupling to the cavity has no effect. Figure 5(c) presents the value of *k*/*k*_0_ from Eq. (36) (scaled by 0.4) as a function of *ω*_c_, depicted by the thick solid lines. A range of light–matter coupling strength *η*_c_ is explored. The FGR expression shows the sharp resonance behavior of the VSC-modified rate profile at *ω*_c_ = *ω*_0_ = 1190 cm^−1^. A similar sharp resonance has been observed in VSC experiments [1], [5], [6] and quantum dynamics simulations [39]. Further, we provide the rate constant calculated from the numerically exact HEOM simulations (see Section VII of the Supplementary Material), depicted by the open circles with a thin guiding line. Although the analytic FGR expression overestimates the rate constant by about two times, the overall agreement between the FGR expression and the HEOM numerical results is remarkable, across the range of *ω*_c_ and *η*_c_ we explored.

Next, we explicitly consider going beyond the single-mode limit. For the 1D FP cavity, $k_{\text{VSC}}^{1\text{D}}$ reduces back to the single-mode approximation. For the 2D FP cavity, based on the expression in Eqs. (13) and (31), the VSC-modified rate constant is expressed as

(40) $$k_{\text{VSC}}^{2\text{D}}=C\cdot g_{\text{c}}^{2}\overset{\omega_{m}}{\underset{\omega_{\text{c}}}{\int }}d\omega \;\frac{\omega {\text{e}}^{-\beta \hbar \omega }}{1+\tau_{\text{c}}/\tau_{\|}\left(\omega \right)}\cdot \frac{\omega^{2}\tau_{\text{c}}^{-1}\omega_{0}\cdot n\left(\omega_{0}\right)}{{\left(\omega^{2}-\omega_{0}^{2}\right)}^{2}+\tau_{\text{c}}^{-2}\omega_{0}^{2}},$$

where $C=\frac{8\pi }{{\left(c\Delta k_{\|}\right)}^{2}Z_{\text{eff}}}$ , and $\tau_{\|}\left(\omega \right)=\omega D/\left[c\sqrt{\omega^{2}-\omega_{\text{c}}^{2}}\;\right]$ (c.f. Eq. (28)). Note that this expression also peaks at *ω*_c_ = *ω*_0_ (as indicated in Figure 4(c)). In Eq. (40), *ω*_c_ is the lower limit of the integral with respect to d*ω*, as well as appearing explicitly in the expression of *τ*_‖_. The result of this definite integral in Eq. (40) is *not* as simple as replacing *ω* with *ω*_c_ as in the single-mode approximation (Eq. (35)).

**Figure 6:**
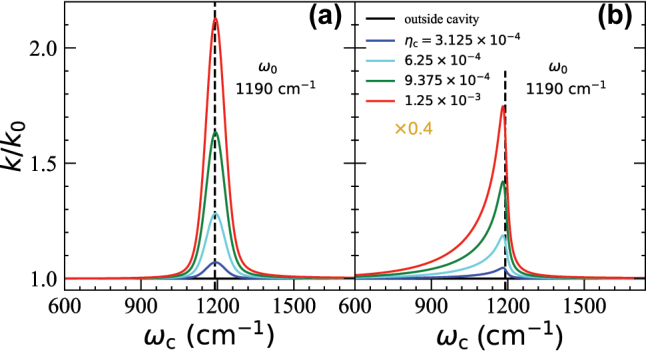
FGR rate profiles of *k*/*k*_0_ as a function of *ω*_c_. (a) FGR rate profiles for the single mode case (or the many modes case inside a 1D FP cavity) calculated using Eq. (36) (same as the solid lines in Figure 5(c)). (b) FGR rate profiles $k_{\text{VSC}}^{2\text{D}}$ for many mode cases inside a 2D FP cavity calculated using Eq. (40). Here, we use D/c=3.33 fs, which corresponds to D=1μ m. Note that both $k_{\text{VSC}}^{1\text{D}}$ and $k_{\text{VSC}}^{2\text{D}}$ are rescaled by a factor of 0.4 to be consistent with Figure 5.

Figure 6 presents the FGR rates under different *η*_c_ values. Figure 6(a) is the same as Figure 5(a), which corresponds to the single-mode case (or the many-mode case inside a 1D FP cavity). Figure 6(b) presents the estimated value of *k*/*k*_0_ using *k*_VSC_ expression in Eq. (40), corresponding to the case of many modes inside a 2D FP cavity. Here, we choose D/c=3.33 fs, corresponding to D=1μ m. This should be viewed as the typical value of D , which is the effective lateral size of a given mode. Results obtained with a range of other choices of D are provided in the Supplementary Material, Section VIII, all of which show a sharp peak at *ω*_c_ ≈ *ω*_0_. Note that the broadening factor (Eq. (36)) was not included for $k_{\text{VSC}}^{2\text{D}}$ for clarity, and one can in principle include it which will further broaden the width of the rate constant distribution. The numerical integration scheme is the same as the calculation of A(ω) , and the convergence is carefully checked. One can observe that the resonance peak is still centered around *ω*_c_ = *ω*_0_ with minor red-shift, which demonstrates the normal incidence condition. The resonance peak is asymmetric due to the asymmetry of A(ω) (see Figure 4(c–f)). Moreover, the rate profile tails toward the lower energy regions, which is the opposite of the trend in A(ω) (see Figure 4). Compared to the single mode version of the theory, considering many modes in a 2D FP cavity predicts that the “action spectrum” of the VSC-modified rate constant has an asymmetric behavior around *ω*_c_ = *ω*_0_, with a longer tail when *ω*_c_ < *ω*_0_. This is an interesting prediction from the current theory in Eq. (40). In recent VSC experiments by Simpkins [8], it does seem that the *ω*_c_ < *ω*_0_ side has a longer tail than the *ω*_c_ > *ω*_0_ side of the action spectrum (*k*_VSC_ vs *ω*_c_ plot, see Figure 3(a) of Ref. [8]). However, this seemingly asymmetrical rate constant profile in Ref. [8] could be caused by a lack of more experimental data points for a blue-tuned cavity (*ω*_c_ > *ω*_0_) due to the experimental difficulty of obtaining such measurements. More experimental data are required to definitively test this trend. Note that in the Simpkins experiment [8] the rate constant was resonantly suppressed. Recent quantum dynamics simulations [39] suggest that by resonantly coupling the cavity mode to a spectator mode (which in turn couples to the reaction coordinate), the rate constant can be suppressed by the cavity. Future work is needed to investigate such a resonance suppression effect.

## 4 Conclusions

We present a theory to explain the current VSC experiments, focusing on the origin of the resonance condition at normal incidence. The theory provides a possible explanation to the resonance condition for the observed VSC effect and of why the resonance effect occurs only at the normal incident angle. In particular, we find that the cavity-modified rate constant *k*_VSC_ can be expressed as the coupling strength multiplied by the accumulated spectral function A(ω) of the cavity, where A(ω) peaks at *ω*_c_ (when *k*_‖_ = 0, *i.e.*, bottom of the dispersion band). For a 1D FP cavity, this is caused by a van-Hove-type singularity (Eq. (24)) in the DOS of the photonic modes. For a 2D FP cavity, we found that one needs to additionally consider the photons propagating outside the mode area associated with *k*_‖_ direction (Eq. (28)), which creates the peak of A(ω) at *ω*_c_. As such, the oblique incidence still has the spectral function peaked at the *ω*_c_, not at the higher incident angle. This theory provides a step forward toward understanding why Rabi splitting is not a *sufficient condition* to achieve a VSC modified rate effect, providing a new insight into the mechanistic understanding of VSC modification.

Under the normal incidence condition, *k*_VSC_ will peak at *ω*_c_ = *ω*_0_. For the 1D cavity case, $k_{\text{VSC}}^{1\text{D}}$ naturally reduces to the single-mode case (Eq. (35)), and we have directly compared the FGR analytic expression with the numerically exact rate constant for a single molecule under strong coupling, which provides agreement across a range of light–matter coupling strengths and cavity frequencies. For the 2D cavity case, we evaluated the FGR rate expression (Eq. (40)), and found a similar sharp resonance at *ω*_c_ = *ω*_0_ compared to the single mode (or the 1D case), with an asymmetric rate constant profile and a long tail when *ω*_c_ < *ω*_0_. This is a unique prediction from the current theory, which should be checked with future experiments.

On the other hand, the current theory cannot explain the observed collective effect, and only when a few molecules are strongly coupled to the cavity can the current theory predict the cavity modifications to the rate constant. This is the limitation of the current theory, and future work is needed to fully address these issues. However, the current work provides significant progress toward building the ultimate theory for understanding VSC effects. Future work will focus on developing a microscopic theory that can explain the collective effect.

## Supplementary Material

See Supplementary Material for additional information on detailed derivations of the Hamiltonian; details of the molecular system; analysis of the Rabi splitting; the effective Hamiltonian and effective spectral density derived by applying harmonic analysis to classical equations of motion; derivation of the VSC-modified rate constant expression in Eq. (13) of the main text; DOS analysis for the 1D and 2D FP cavity; details of the quantum dynamics simulation results; effects of the D/c values on the rate profiles for the 2D cavity case.

## Supplementary Material

Supplementary Material Details
