# Supplementary material for: Resonance theory of vibrational polariton chemistry at the normal incidence
Source: Nanophotonics. 2024 Feb 23;13(14):2601–15. doi: 10.1515/nanoph-2023-0685 (PMC11636501; doi:10.1515/nanoph-2023-0685)
Supplement: Supplementary file 1 — Supplementary Material Details [file j_nanoph-2023-0685_suppl_001.pdf]

Supplementary Material for
Resonance Theory of Vibrational Polariton Chemistry at the Normal Incidence

Wenxiang Ying,[1,*] Michael A.D. Taylor,[2] and Pengfei Huo[1,2,†]

[1] *Department of Chemistry, University of Rochester, 120 Trustee Road, Rochester, New York 14627, USA*
[2] *Institute of Optics, Hajim School of Engineering, University of Rochester, Rochester, New York 14627, USA*

CONTENTS

I. Details of the Hamiltonian S1
II. Details of the Molecular Model System S4
III. Analysis of the Rabi Splitting S5
IV. The effective Hamiltonian and spectral density S6
V. Derivation of the FGR rate under many-modes cases S8
VI. The density of states and the effective spectral density S9
VII. Quantum Dynamics Simulations of the lossy cavity, and the rate constant S11
VIII. Effects of the $\mathcal{D}/c$ values on the rate profiles for the 2D cavity case S13
References S13

I. DETAILS OF THE HAMILTONIAN

We start with the Pauli-Fierz Hamiltonian of many molecules coupled to many modes under the dipole approximation. This Hamiltonian is obtained by performing the Power-Zienau-Woolley (PZW) gauge transformation [1–3] on the minimum coupling Hamiltonian. The details can be found in Ref. 4 (Sec. 2.6) or Ref. 5 (Chapter 2.2). The Hamiltonian is then further projected on the ground electronic states of all molecules.

The total Hamiltonian is expressed as

$$\begin{aligned}\hat{H} &= \hat{H}_{\mathrm{M}} + \hat{H}_{\mathrm{ph}} + \hat{H}_{\mathrm{loss}} \\ &+ \sum_{\mathbf{k}}\Big[\sqrt{\frac{\hbar\omega_{\mathbf{k}}}{2}}\lambda_{\mathrm{c}}\sum_{j}(\hat{a}^{\dagger}_{\mathbf{k}}e^{-i\mathbf{k}\cdot\bar{\boldsymbol{x}}_j} + \hat{a}_{\mathbf{k}}e^{i\mathbf{k}\cdot\bar{\boldsymbol{x}}_j})(\hat{\mathrm{e}}_{\mathbf{k}}\cdot\hat{\boldsymbol{\mu}}(\hat{R}_j)) + \frac{\lambda_{\mathrm{c}}^2}{2}\sum_{i,j}(\hat{\mathrm{e}}_{\mathbf{k}}\cdot\hat{\boldsymbol{\mu}}(\hat{R}_i))(\hat{\mathrm{e}}_{\mathbf{k}}\cdot\hat{\boldsymbol{\mu}}(\hat{R}_j))e^{-i\mathbf{k}\cdot(\bar{\boldsymbol{x}}_i-\bar{\boldsymbol{x}}_j)}\Big],\end{aligned} \tag{S1}$$

where the $\{i,j\}$ iterates over the molecules in the cavity, $\bar{\boldsymbol{x}}_j$ is the center of mass of the j_{th} molecule, $\hat{H}_{\mathrm{M}}$ is the bare matter Hamiltonian, $\hat{H}_{\mathrm{ph}}$ is the pure photonic Hamiltonian. Further, $\hat{H}_{\mathrm{M}}$ is the molecular Hamiltonian

$$\hat{H}_{\mathrm{M}} = \sum_{j=1}^{N}\Big(\frac{\hat{P}_j^2}{2M} + V(\hat{R}_j)\Big) + \hat{H}_{\nu}, \tag{S2}$$

where $\hat{R}_j$ is the reaction coordinate for the j_{th} molecule, $V(\hat{R})$ is the common ground state potential for all reaction molecules (typically double well potential), and $\hat{\boldsymbol{\mu}}(\hat{R}_j)$ is the dipole operator associated with the ground electronic

* wying3@ur.rochester.edu
† pengfei.huo@rochester.edu

state of reaction coordinate $\hat{R}_j$ (electronic permanent dipole). In this work, we have explicitly ignored the interactions among molecules and treated them as independent, identical molecules. We further introduce the effects of a phonon bath to the Hamiltonian via a system-bath term, which reads as

$$\hat{H}_\nu = \frac{1}{2}\sum_j\sum_\zeta \Big[\hat{p}_{j,\zeta}^2 + \omega_{j,\zeta}^2\Big(\hat{x}_{j,\zeta} - \frac{c_{j,\zeta}}{\omega_{j,\zeta}^2}\hat{R}_j\Big)^2\Big], \tag{S3}$$

where $\{\hat{x}_{j,\zeta}, \hat{p}_{j,\zeta}\}$ are the mass-weighted coordinate and momentum pair of the $\{j,\zeta\}$-th bath oscillator that directly couples to the reaction coordinate of molecule j. The j-th phonon bath as well as its coupling to the reaction coordinate R_j can be described by the spectral density function [6]

$$J_\nu(\omega) = \frac{\pi}{2}\sum_j\sum_\zeta \frac{c_{j,\zeta}^2}{\omega_{j,\zeta}}\delta(\omega - \omega_{j,\zeta}), \tag{S4}$$

where $\omega_{j,\zeta}$, $c_{j,\zeta}$ are the oscillator frequencies and coupling coefficients, respectively. Note that we have assumed an identical spectral density for all molecule $j \in [1, N]$.

By introducing the photon mode coordinate and momentum operators

$$\hat{q}_{\mathbf{k}} = \sqrt{\hbar/2\omega_{\mathbf{k}}}(\hat{a}_{\mathbf{k}}^\dagger + \hat{a}_{\mathbf{k}}), \qquad \hat{p}_{\mathbf{k}} = i\sqrt{\hbar\omega_{\mathbf{k}}/2}(\hat{a}_{\mathbf{k}}^\dagger - \hat{a}_{\mathbf{k}}), \tag{S5}$$

or inversely, the field operators

$$\hat{a}_{\mathbf{k}} = \sqrt{\frac{\omega_{\mathbf{k}}}{2\hbar}}\hat{q}_{\mathbf{k}} + i\sqrt{\frac{1}{2\hbar\omega_{\mathbf{k}}}}\hat{p}_{\mathbf{k}}, \qquad \hat{a}_{\mathbf{k}}^\dagger = \sqrt{\frac{\omega_{\mathbf{k}}}{2\hbar}}\hat{q}_{\mathbf{k}} - i\sqrt{\frac{1}{2\hbar\omega_{\mathbf{k}}}}\hat{p}_{\mathbf{k}}, \tag{S6}$$

Eq. S1 can be alternatively expressed as

$$\hat{H} = \hat{H}_{\mathrm{M}} + \frac{1}{2}\sum_{\mathbf{k}}\Big[(\hat{p}_{\mathbf{k}} - \lambda_{\mathrm{c}}\hat{\Pi}_{\mathbf{k}})^2 + (\omega_{\mathbf{k}}\hat{q}_{\mathbf{k}} + \lambda_{\mathrm{c}}\hat{\mathcal{S}}_{\mathbf{k}})^2\Big] + \hat{H}_{\mathrm{loss}}, \tag{S7}$$

where the collective system operators are defined as

$$\hat{\Pi}_{\mathbf{k}} = \sum_j(\hat{\boldsymbol{\mu}}(\hat{R}_j)\cdot\hat{\mathrm{e}}_{\mathbf{k}})\sin(\mathbf{k}\cdot\bar{\boldsymbol{x}}_j), \tag{S8a}$$

$$\hat{\mathcal{S}}_{\mathbf{k}} = \sum_j(\hat{\boldsymbol{\mu}}(\hat{R}_j)\cdot\hat{\mathrm{e}}_{\mathbf{k}})\cos(\mathbf{k}\cdot\bar{\boldsymbol{x}}_j). \tag{S8b}$$

To account for cavity loss, we further introduce the photon-loss Hamiltonian which is also based on the system-bath model, defined as

$$\hat{H}_{\mathrm{loss}} = \frac{1}{2}\sum_{\mathbf{k},\zeta}\Big[\hat{p}_{\mathbf{k},\zeta}^2 + \omega_{\mathbf{k},\zeta}^2\Big(\hat{x}_{\mathbf{k},\zeta} - \frac{c_{\mathbf{k},\zeta}}{\omega_{\mathbf{k},\zeta}^2}\hat{q}_{\mathbf{k}}\Big)^2\Big], \tag{S9}$$

where $\{\hat{x}_{\mathbf{k},\zeta}, \hat{p}_{\mathbf{k},\zeta}\}$ are the mass-weighted coordinate and momentum operators of the $\{\mathbf{k},\zeta\}$-th non-cavity bath mode, respectively, which directly couple to the photon mode coordinate operator $\hat{q}_{\mathbf{k}}$. The $\mathbf{k}$-th photon-loss bath as well as its coupling to the photon mode coordinate operator $\hat{q}_{\mathbf{k}}$ are described by the spectral density function

$$J_{\mathrm{loss}}(\omega,\mathbf{k}) = \frac{\pi}{2}\sum_\zeta \frac{c_{\mathbf{k},\zeta}^2}{\omega_{\mathbf{k},\zeta}}\delta(\omega - \omega_{\mathbf{k},\zeta}), \tag{S10}$$

where $\omega_{\mathbf{k},\zeta}$, $c_{\mathbf{k},\zeta}$ are the oscillator frequencies and coupling coefficients, respectively.

Based on the above discussions, the collective PF Hamiltonian in Eq. S7 is expressed in the projected subspace as

$$\hat{H} = \sum_j \frac{\hat{P}_j}{2M} + V(\hat{R}_j) + \hat{H}_\nu + \frac{1}{2}\sum_{\mathbf{k}}\Big[(\hat{p}_{\mathbf{k}} - \lambda_{\mathrm{c}}\hat{\Pi}_{\mathbf{k}})^2 + (\omega_{\mathbf{k}}\hat{q}_{\mathbf{k}} + \lambda_{\mathrm{c}}\hat{\mathcal{S}}_{\mathbf{k}})^2\Big] + \hat{H}_{\mathrm{loss}}, \tag{S11}$$

where $\hat{\Pi}_{\mathbf{k}}$ and $\hat{\mathcal{S}}_{\mathbf{k}}$ are defined in Eq. S8.

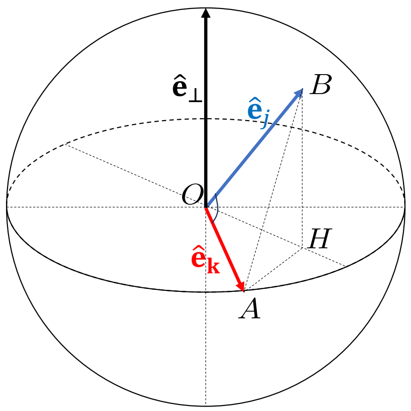

FIG. S1. Schematic illustration about orientations of the dipole operator and field polarization vector on a unit sphere, and their geometric relations.

For simplicity, in this work we assume the *long wavelength approximation* where the transverse fields can be treated as spatially uniform, *i.e.*, $e^{i\mathbf{k}\cdot\mathbf{r}} \approx 1$, such that

$$\hat{\mathbf{A}}_\perp(\mathbf{r}) \approx \hat{\mathbf{A}}_\perp = \sum_\mathbf{k} \frac{\hat{\mathrm{e}}_\mathbf{k}}{\omega_\mathbf{k}} \sqrt{\frac{\hbar\omega_\mathbf{k}}{2\varepsilon_0 \mathcal{V}}} (\hat{a}_\mathbf{k} + \hat{a}^\dagger_\mathbf{k}), \tag{S12}$$

which leads to $\cos(\mathbf{k}\cdot\bar{\boldsymbol{x}}_j) = 1$, $\sin(\mathbf{k}\cdot\bar{\boldsymbol{x}}_j) = 0$, $\hat{\Pi}_\mathbf{k} = 0$ and $\hat{\mathcal{S}}_\mathbf{k} = \sum_j \hat{\boldsymbol{\mu}}(\hat{R}_j)\cdot\hat{\mathrm{e}}_\mathbf{k}$. Then the collective PF Hamiltonian of Eq. S11 can be further simplified as

$$\hat{H} = \sum_j \frac{\hat{P}_j}{2M} + V(R_j) + \hat{H}_\nu + \frac{1}{2}\sum_\mathbf{k}\left[\hat{p}_\mathbf{k}^2 + \omega_\mathbf{k}^2\Big(\hat{q}_\mathbf{k} + \frac{\lambda_\mathrm{c}}{\omega_\mathbf{k}}\sum_j \hat{\boldsymbol{\mu}}(\hat{R}_j)\cdot\hat{\mathrm{e}}_\mathbf{k}\Big)^2\right] + \hat{H}_\mathrm{loss}, \tag{S13}$$

which is the VSC Hamiltonian in Eq. 1 of the main text.

We further define $\varphi_{j,\mathbf{k}}$ as the angle between $\hat{\boldsymbol{\mu}}(\hat{R}_j)$ (the dipole operator of the j_th molecule) and the field polarization direction $\hat{\mathbf{e}}_\mathbf{k}$, where we only consider transverse electric (TE) polarization. As a result, $\hat{\mathbf{e}}_\mathbf{k}$ is always parallel to the longitudinal direction, and $\hat{\boldsymbol{\mu}}(\hat{R}_j)\cdot\hat{\mathbf{e}}_\mathbf{k} = \mu(\hat{R}_j)\cos\varphi_{j,\mathbf{k}}$, where $\hat{\boldsymbol{\mu}}(\hat{R}_j) = \mu(\hat{R}_j)\cdot\hat{\mathbf{e}}_j$ is the ground state permanent dipole moment of molecule j, and $\hat{\mathbf{e}}_j$ is the unit vector that parallel to the dipole vector. A schematic illustration is provided in Fig. S1 below, where we consider a 2D FP cavity and use a unit sphere to schematically illustrate orientations of the dipole and field polarization vector, as well as their geometric relations. In Fig. S1, point O is the center of the unit sphere, $\hat{\mathbf{e}}_\perp$ denotes the perpendicular direction (or the quantized direction). Since we only consider the TE polarization of the cavity modes, the electric field polarization direction, which is described by unit vector $\vec{OA} \equiv \hat{\mathbf{e}}_\mathbf{k}$ where point A is on the equator of the unit sphere, is always perpendicular to $\hat{\mathbf{e}}_\perp$, *i.e.*, $\hat{\mathbf{e}}_\perp\cdot\hat{\mathbf{e}}_\mathbf{k} = 0$. As a result, $\hat{\mathbf{e}}_\mathbf{k}$ determines a plane that is perpendicular to $\hat{\mathbf{e}}_\perp$ and parallel to the cavity mirrors. Further, $\vec{OB} \equiv \hat{\mathbf{e}}_j$ denotes the unit vector that is parallel to the dipole orientation, where point B is arbitrary on the unit sphere. Angle $\varphi_{j,\mathbf{k}}$ is then determined by $\angle AOB$. Suppose point H is the projection of point B on the plane determined by $\hat{\mathbf{e}}_\mathbf{k}$, *i.e.*, $\vec{BH}$ is parallel to $\hat{\mathbf{e}}_\perp$. We further define $\varphi_j = \angle BOH$ which characterizes the angle between the dipole and the plane that varies from 0 to π, and $\phi_{j,\mathbf{k}} = \angle AOH$ which characterizes the angle between the field polarization and the projection of the dipole on the plane that varies from 0 to 2π. It is easy to show that

$$\cos\varphi_{j,\mathbf{k}} = \cos\varphi_j \cdot \cos\phi_{j,\mathbf{k}}. \tag{S14}$$

For 1D FP cavity case, $\varphi_{j,\mathbf{k}}$ will reduce to φ_j since $\phi_{j,\mathbf{k}} = 0$.

To summarize, we have explicitly assumed that there are no intermolecular interactions, as well as the long wavelength approximation. Their presence might influence the final rate constant expression k_VSC, however, they are not playing any dominant role in order to provide a theory as we have shown here to capture resonant effect and collective effect.

II. DETAILS OF THE MOLECULAR MODEL SYSTEM

To model how VSC influences chemical reactions, we are particularly interested in the one-dimensional double-well (DW) potential [7, 8]

$$V(\hat{R}) = -\frac{M\omega_{\rm b}^2}{2}\hat{R}^2 + \frac{M^2\omega_{\rm b}^4}{16E_{\rm b}}\hat{R}^4, \tag{S15}$$

where M is the effective mass of the reaction coordinate, $\omega_{\rm b}$ is the barrier frequency, and $E_{\rm b}$ is barrier height of the DW potential. Note that Eq. S15 assumes a symmetric DW potential.

For the system (reaction coordinate), the corresponding eigenvectors $|\nu_i\rangle$ and eigenenergies E_i are obtained by numerically solving

$$\Big(\frac{\hat{P}^2}{2M} + V(\hat{R})\Big)|\nu_i\rangle = E_i|\nu_i\rangle, \tag{S16}$$

where $V(\hat{R})$ is expressed in Eq. S15. These vibrational eigenstates are obtained by using the discrete variable representation (DVR) basis [9]. We *diabatize* the two lowest eigenstates as

$$|\nu_{\rm L}\rangle = \frac{1}{\sqrt{2}}\big(|\nu_0\rangle + |\nu_1\rangle\big), \qquad |\nu_{\rm R}\rangle = \frac{1}{\sqrt{2}}\big(|\nu_0\rangle - |\nu_1\rangle\big), \tag{S17}$$

which leads to two energetically degenerate diabatic states, denoted as $|\nu_{\rm L}\rangle$ and $|\nu_{\rm R}\rangle$ for states localized in the left and right wells, respectively, both with the degenerate energy $E_{\rm L} = (E_1 + E_0)/2$ and a small tunneling splitting $V_{\rm LR}^0 = (E_1 - E_0)/2$ (where the energy difference between E_1 and E_0 is $2V_{\rm LR}^0$). Similarly, for $\{|\nu_2\rangle, |\nu_3\rangle\}$, one can diabatize them and obtain the first excited *diabatic vibrational state* in the left well and right well as follows

$$|\nu'_{\rm L}\rangle = \frac{1}{\sqrt{2}}\big(|\nu_2\rangle + |\nu_3\rangle\big), \qquad |\nu'_{\rm R}\rangle = \frac{1}{\sqrt{2}}\big(|\nu_2\rangle - |\nu_3\rangle\big), \tag{S18}$$

with the degenerate diabatic energy $E_{\rm L'} = (E_3 + E_2)/2$ and the tunneling splitting $V_{\rm LR} = (E_3 - E_2)/2$. Based on the two diabatic states $|\nu_{\rm L}\rangle$ and $|\nu'_{\rm L}\rangle$ in the left well, we define the quantum vibration frequency of the reactant as

$$\hbar\omega_0 \equiv E_{\rm L'} - E_{\rm L}, \tag{S19}$$

which is directly related to the quantum transition of $|\nu_{\rm L}\rangle \to |\nu'_{\rm L}\rangle$. Note that the spectroscopy measurement (IR or transmission spectra) is also directly related to this frequency.

For a practical calculation, truncation has to be made upon the number of matter states, restricting the dynamics in a relatively low energy subspace while ensuring numerical accuracy. As such, the Hamiltonian and the reaction coordinate have their matrix representations in a truncated Hilbert space. Similarly, we also have the vibrational permanent dipole associated with $|\nu_{\rm L}\rangle$ as $\mu_{\rm LL} = \langle\nu_{\rm L}|\mu(\hat{R})|\nu_{\rm L}\rangle$, as well as for vibrationally excited states $|\nu'_{\rm L}\rangle$ as $\mu_{\rm L'L'} = \langle\nu'_{\rm L}|\mu(\hat{R})|\nu'_{\rm L}\rangle$. These permanent dipoles might be important for computing polariton spectra under ultra-strong coupling regimes. For rate constants, we find that they might be important to provide constant shifts of vibrational states under very large coupling limits. We have ignored them for the simplicity of the theory.

Fig. 2a of the main text provides a schematic illustration of the ground state chemical reaction model (single molecule) and the first few vibrational states of the DW model, denoted as $|\nu_{\rm L}\rangle$, $|\nu_{\rm R}\rangle$, $|\nu'_{\rm L}\rangle$, $|\nu'_{\rm R}\rangle$. The red arrows indicate the potential effect of the cavity modifying vibrational state transitions, and the green arrow right above the barrier denotes to the fast dissipative tunneling process from $|\nu'_{\rm L}\rangle$ to $|\nu'_{\rm R}\rangle$. Here, we use the parameters $E_{\rm b} = 2250$ cm^{-1}, and $\hbar\omega_{\rm b} = 1000$ cm^{-1}. [10] The eigenstates are obtained with the sinc-DVR basis with 1001 grid points in the range of $-100 \leq R \leq 100$, then diabatized according to Eq. S17 and S18. Note that because $|\nu'_{\rm L}\rangle$ and $|\nu'_{\rm R}\rangle$ are very close to the top of the barrier, they are not as well localized as $|\nu_{\rm L}\rangle$ and $|\nu_{\rm R}\rangle$. To be more clear, we briefly summarize the major parameters for the system degrees of freedom (DOFs) in Table S1. [10]

We further show that the subsequent step $\{|\nu'^j_{\rm L}\rangle\} \xrightarrow{k_2} \{|\nu'^j_{\rm R}\rangle\}$ will occur with the same rate constant k_2 as for the cavity free case, such that there is *no additional* change of this step due to coupling to the cavity. Note that outside the cavity, this rate is controlled by the tunneling-splitting coupling between $|\nu'^j_{\rm L}\rangle$ and $|\nu'^j_{\rm R}\rangle$, denoted as $V_{\rm LR} = \langle\nu'^j_{\rm R}|\hat{V}_j|\nu'^j_{\rm L}\rangle$, which is assumed to be identical for all molecules j. The localness of chemical reactions ensures that $\langle\nu'^i_{\rm R}|\hat{V}_j|\nu'^k_{\rm L}\rangle = V_{\rm LR}\delta_{ij}\delta_{jk}$, *i.e.*, the reaction occurs locally. Using similar Fermi's Golden Rule (FGR) argument for the $\{|\nu'^j_{\rm L}\rangle\} \xrightarrow{k_2} \{|\nu'^j_{\rm R}\rangle\}$ transition, We assume that by reaching the steady state populations, all $|\nu'^j_{\rm L}\rangle$ are equally

TABLE S1. Table of major parameters

Parameters of system DOFs	Notation	Value
Effective mass of the reaction coordinate	M	1 a.u.
Barrier height	E_{b}	2250 cm^{-1}
Barrier frequency	$\hbar\omega_{\mathrm{b}}$	1000 cm^{-1}
Vibration frequency (or resonance frequency)	$\hbar\omega_0$	1190 cm^{-1}
Tunneling splitting between $\vert\nu_{\mathrm{L}}\rangle$ and $\vert\nu_{\mathrm{R}}\rangle$	V_{LR}^0	1.03 cm^{-1}
Tunneling splitting between $\vert\nu'_{\mathrm{L}}\rangle$ and $\vert\nu'_{\mathrm{R}}\rangle$	V_{LR}	47.68 cm^{-1}

populated, such that each channel should be weighted by $1/N$. Using FGR, and considering a resonant energy tunneling transition between $|\nu'^{j}_{\mathrm{L}}\rangle$ and $|\nu'^{j}_{\mathrm{R}}\rangle$, the tunneling rate constant k_2 is

$$k_2 \propto \frac{1}{N}\sum_{j=1}^{N}|\langle\nu'^{j}_{\mathrm{R}}|\hat{V}_j|\nu'^{j}_{\mathrm{L}}\rangle|^2 \propto V_{\mathrm{RL}}^2, \tag{S20}$$

indicating that k_2 is identical to the single molecule tunneling rate outside the cavity. As such, the molecule-cavity interaction will not change the local bond-breaking process.

III. ANALYSIS OF THE RABI SPLITTING

To quickly review the well-known results of Rabi splitting through collective light-matter couplings, let us assume all dipoles are fully aligned to the field polarization direction, such that $\cos\varphi_{j,\mathbf{k}} = 1$ for all j and a given $\mathbf{k}$. We further introduce the $\sigma_j = |G\rangle\langle\nu_j|$ and $\sigma_j^\dagger = |\nu_i\rangle\langle G|$ as the raising and lowering operators of the molecular vibrational excitation on molecule j. In the single excited subspace $\mathcal{P} = |G\rangle\langle G| + \sum_j |\nu_j\rangle\langle\nu_j|$, the $\mu(\hat{R}_j)$ operator becomes

$$\hat{\mathcal{P}}\mu(\hat{R}_j)\hat{\mathcal{P}} = \mu_{\mathrm{LL'}}\cdot(\sigma_j^\dagger + \sigma_j), \tag{S21}$$

where $\mu_{\mathrm{LL'}} = \langle\nu'^{j}_{\mathrm{L}}|\mu(\hat{R}_j)|\nu^{j}_{\mathrm{L}}\rangle$ is identical to all molecules. Here, we explicitly ignored the permanent dipole contributions $\langle\nu^{j}_{\mathrm{L}}|\hat{R}_j|\nu^{j}_{\mathrm{L}}\rangle$ and $\langle\nu'^{j}_{\mathrm{L}}|\hat{R}_j|\nu'^{j}_{\mathrm{L}}\rangle$, which for our model have very small matrix elements. They could be important for computing the polariton eigenspectrum when the coupling strength λ_{c} is very large.

Using the above notations, one can rewrite the light-matter coupling term as

$$\hat{H}_{\mathrm{LM}}^{[N]} = \hbar g_{\mathrm{c}}\sqrt{\omega_{\mathbf{k}}}\cdot\sum_j\sum_{\mathbf{k}}(\hat{\sigma}_j + \hat{\sigma}_j^\dagger)(\hat{a}_{\mathbf{k}} + \hat{a}_{\mathbf{k}}^\dagger), \tag{S22}$$

where $g_{\mathrm{c}} = \mu_{\mathrm{LL'}}\lambda_{\mathrm{c}}/\sqrt{2\hbar}$. When assuming $\cos\varphi_{j,\mathbf{k}} = 1$, Eq. S13 will have permutation symmetry, and one can introduce the collective operators

$$\hat{\sigma}_N^\dagger = \frac{1}{\sqrt{N}}\sum_j |\nu_j\rangle\langle G|, \quad \hat{\sigma}_N = \frac{1}{\sqrt{N}}\sum_j |G\rangle\langle\nu_j|. \tag{S23}$$

Using the collective operators, and ignoring the counter-rotating wave term ($\propto \sum_{j,\mathbf{k}}(\hat{\sigma}_j^\dagger\hat{a}_{\mathbf{k}}^\dagger + \hat{\sigma}_j\hat{a}_{\mathbf{k}})$), which is less important for the resonant condition of $\omega_{\mathbf{k}} = \omega_0$ when g_{c} is small, we have

$$\hat{H}_{\mathrm{LM}}^{[N]} = \sqrt{N}\hbar g_{\mathrm{c}}\sqrt{\omega_{\mathbf{k}}}\cdot\sum_{\mathbf{k}}(\sigma_N^\dagger\hat{a}_{\mathbf{k}} + \sigma_N\hat{a}_{\mathbf{k}}^\dagger). \tag{S24}$$

This level of approximation is commonly referred to as the Tavis-Cummings model. Note that the effective coupling between light and matter is now $\sqrt{N}g_{\mathrm{c}}$. This light-matter coupling term will hybridize the 1 photon-dressed ground state $|G\rangle\otimes|1_{\mathbf{k}}\rangle$ with the 0-photon dressed bright state $|B\rangle\otimes|0_{\mathbf{k}}\rangle$, generating the following polariton states

$$|+\rangle = \cos\phi_N\cdot|\mathrm{B}\rangle\otimes|0_{\mathbf{k}}\rangle + \sin\phi_N\cdot|G\rangle\otimes|1_{\mathbf{k}}\rangle, \tag{S25a}$$

$$|-\rangle = -\sin\phi_N\cdot|\mathrm{B}\rangle\otimes|0_{\mathbf{k}}\rangle + \cos\phi_N\cdot|G\rangle\otimes|1_{\mathbf{k}}\rangle, \tag{S25b}$$

where the mixing angle, ϕ_N, is defined as

$$\phi_N = \frac{1}{2}\tan^{-1}[(2\sqrt{N\omega_{\mathbf{k}}}g_{\mathrm{c}})/(\omega_{\mathbf{k}}(k_{\|}) - \omega_0)], \tag{S26}$$

with the maximum mixing between light and matter occuring when $\omega_{\mathrm{c}}(\mathbf{k}) = \omega_0$. The dark states, on the other hand, do not mix with the photonic DOF under this approximation and remain to be $|\mathrm{D}_\alpha\rangle \otimes |0_{\mathbf{k}}\rangle$ in the singly excited subspace. It is a well-known fact that $\langle G| \otimes \langle 1_{\mathbf{k}}|\hat{H}_{\mathrm{LM}}|\mathrm{D}_\alpha\rangle \otimes |0_{\mathbf{k}}\rangle = 0$, because $\langle G|\sum_j(\sigma_j + \sigma_j^\dagger)|\sum_k \mathcal{C}_k^\alpha|\nu_k\rangle = \sum_j\langle\nu_j|\sum_k \mathcal{C}_k^\alpha|\nu_k\rangle = \sum_k \mathcal{C}_k^\alpha = 0$. As such, these states are *dark* because they do not contain photonic components, but also there is no optical transition to them from $|G\rangle$. The energy gap between the upper polariton state and the lower polariton state is referred to as the Rabi splitting and is expressed as follows

$$\Omega_{\mathrm{R}} \equiv E_+ - E_- = \sqrt{(\omega_{\mathbf{k}} - \omega_0)^2 + 4N\omega_{\mathrm{c}}g_{\mathrm{c}}^2}, \tag{S27}$$

and under the resonant condition $\omega_{\mathbf{k}} = \omega_0$ with a given $\mathbf{k}$, the Rabi splitting is $\Omega_{\mathrm{R}} = 2\sqrt{N\omega_{\mathrm{c}}}g_{\mathrm{c}} = \sqrt{\frac{2\omega_{\mathbf{k}}}{\epsilon_0\hbar}}\sqrt{\frac{N}{\mathcal{V}}}\cdot\mu_{\mathrm{LL}'}$. As one can clearly see, forming the Rabi splitting is originated from a collective phenomenon, resulting in the well-known $\sqrt{N}$ dependence or $\sqrt{N/\mathcal{V}}$ dependence (square root of concentration), confirmed by experiments [11].

IV. THE EFFECTIVE HAMILTONIAN AND SPECTRAL DENSITY

Recall the total Hamiltonian in Eq. S13) and separate the $\mathbf{k}$-dependent and $\mathbf{k}$-independent components by using the relation $\cos\varphi_{j,\mathbf{k}} = \cos\varphi_j \cdot \cos\phi_{j,\mathbf{k}}$, and with the assumption that $\phi_{j,\mathbf{k}} \to \phi_{\mathbf{k}}$ is j-independent for the 2D cavity case, we have

$$\hat{H} = \sum_{j=1}^{N}\left[\frac{\hat{P}_j^2}{2M} + V(\hat{R}_j)\right] + \hat{H}_\nu + \frac{1}{2}\sum_{\mathbf{k}}\left[\hat{p}_{\mathbf{k}}^2 + \omega_{\mathbf{k}}^2\Big(\hat{q}_{\mathbf{k}} + \frac{\lambda_{\mathrm{c}}}{\omega_{\mathbf{k}}}\cos\phi_{\mathbf{k}}\sum_{j=1}^{N}\mu(\hat{R}_j)\cdot\cos\varphi_j\Big)^2\right] + \hat{H}_{\mathrm{loss}}. \tag{S28}$$

It is shown that the model Hamiltonian has a one-to-one map (through normal mode transformation) to the effective Hamiltonian as below, [12, 13]

$$\hat{H} = \sum_{j=1}^{N}\left[\frac{\hat{P}_j^2}{2M} + V(\hat{R}_j)\right] + \hat{H}_\nu + \frac{1}{2}\sum_{\mathbf{k},\zeta}\left[\hat{\tilde{p}}_j^2 + \tilde{\omega}_{\mathbf{k},\zeta}^2\Big(\hat{\tilde{x}}_{\mathbf{k},\zeta} - \frac{\tilde{c}_{\mathbf{k},\zeta}}{\tilde{\omega}_{\mathbf{k},\zeta}^2}\sum_{j=1}^{N}\mu(\hat{R}_j)\cdot\cos\varphi_j\Big)^2\right], \tag{S29}$$

The effective bath $\{\hat{\tilde{x}}_{\mathbf{k}}\}$ and its interaction with the system dissipation modes are described by effective spectral density functions as follows

$$J_{\mathrm{eff}}(\omega_{\mathbf{k}}, \omega) \equiv \frac{1}{2}\int_{-\infty}^{\infty} dt\; e^{i\omega t}\langle[\hat{F}_{\mathbf{k}}(t), \hat{F}_{\mathbf{k}}(0)]\rangle_{\mathrm{B}} = \frac{\pi}{2}\sum_{\mathbf{k},\zeta}\frac{\tilde{c}_{\mathbf{k},\zeta}^2}{\tilde{\omega}_{\mathbf{k},\zeta}}\delta(\omega - \tilde{\omega}_{\mathbf{k},\zeta}), \tag{S30}$$

where the stochastic force exerted by the $\mathbf{k}$-th effective bath is

$$\hat{F}_{\mathbf{k}} = \cos\phi_{\mathbf{k}}\cdot\sum_{\zeta}\tilde{c}_{\mathbf{k},\zeta}\hat{\tilde{x}}_{\mathbf{k},\zeta}, \tag{S31}$$

and $\langle\cdot\rangle_{\mathrm{B}} \equiv \mathrm{Tr}_{\mathrm{B}}[\;\cdot\;\hat{\rho}_{\mathrm{B}}^{\mathrm{eq}}]$, where $\hat{\rho}_{\mathrm{B}}^{\mathrm{eq}}$ is the bath density operator under thermal equilibrium. Note that the effective baths serve as the common bath for all the system DOFs $\hat{R}_j$.

Below, we provide a concise proof for Eq. S29 and derive the explicit expression of the effective spectral density function. For convenience, we denote $\hat{\mathcal{S}} = \sum_{j=1}^{N}\mu(\hat{R}_j)\cdot\cos\varphi_j$ as the collective system dipole operator. The terms in the total Hamiltonian in Eq. S28 for this normal mode transformation is

$$\hat{H} - \hat{H}_\nu = \sum_{j=1}^{N}\left[\frac{\hat{P}_j^2}{2M} + V(\hat{R}_j)\right] + \frac{1}{2}\sum_{\mathbf{k}}\left[\hat{p}_{\mathbf{k}}^2 + \omega_{\mathbf{k}}^2\left(\hat{q}_{\mathbf{k}} + \frac{\lambda_{\mathrm{c}}\cos\phi_{\mathbf{k}}}{\omega_{\mathbf{k}}}\hat{\mathcal{S}}\right)^2\right]$$
$$+ \frac{1}{2}\sum_{\mathbf{k},\zeta}\left[\hat{\tilde{p}}_{\mathbf{k},\zeta}^2 + \tilde{\omega}_{\mathbf{k},\zeta}^2\left(\hat{\tilde{x}}_{\mathbf{k},\zeta} - \frac{\tilde{c}_{\mathbf{k},\zeta}}{\tilde{\omega}_{\mathbf{k},\zeta}^2}\hat{q}_{\mathbf{k}}\right)^2\right], \tag{S32}$$

where $\hat{H}_\nu$ (system-phonon interaction) is omitted, because it is not directly involved in light-matter interactions. Next, by applying harmonic analysis to the equations of motion, we derive the effective spectral density function which describes the cavity modes as well as their associated loss. We will follow and generalize the approach proposed by Leggett [14] and Garg, *et al.* [12]. The classical equations of motion with respect to the Hamiltonian in Eq. S32 can be formally written down as

$$\ddot{\mathcal{S}} = -\frac{\partial V(\{R_j\})}{\partial \mathcal{S}} - \sum_{\mathbf{k}} \omega_{\mathbf{k}}^2 \cdot \frac{\lambda_c \cos\phi_{\mathbf{k}}}{\omega_{\mathbf{k}}} \left(q_{\mathbf{k}} + \frac{\lambda_c \cos\phi_{\mathbf{k}}}{\omega_{\mathbf{k}}} \mathcal{S} \right), \tag{S33a}$$

$$\ddot{q}_{\mathbf{k}} = -\omega_{\mathbf{k}}^2 \left(q_{\mathbf{k}} + \frac{\lambda_c \cos\phi_{\mathbf{k}}}{\omega_{\mathbf{k}}} \mathcal{S} \right) + \sum_{\zeta} \left(\tilde{c}_{\mathbf{k},\zeta} \tilde{x}_{\mathbf{k},\zeta} - \frac{\tilde{c}_{\mathbf{k},\zeta}^2}{\tilde{\omega}_{\mathbf{k},\zeta}^2} q_{\mathbf{k}} \right), \tag{S33b}$$

$$\ddot{\tilde{x}}_{\mathbf{k},\zeta} = -\tilde{\omega}_{\mathbf{k},\zeta}^2 \tilde{x}_{\mathbf{k},\zeta} + \tilde{c}_{\mathbf{k},\zeta} q_{\mathbf{k}}. \tag{S33c}$$

Applying Fourier transform to Eq. S33 leads to

$$\left(-\omega^2 + \sum_{\mathbf{k}} \lambda_c^2 \cos^2\phi_{\mathbf{k}} \right) \mathcal{S}(\omega) + \sum_{\mathbf{k}} \lambda_c \cos\phi_{\mathbf{k}} \omega_{\mathbf{k}} q_{\mathbf{k}}(\omega) = -V'_\omega, \tag{S34a}$$

$$\left[(\omega_{\mathbf{k}}^2 - \omega^2) + \sum_{j} \frac{\tilde{c}_{\mathbf{k},\zeta}^2}{\tilde{\omega}_{\mathbf{k},\zeta}^2} \right] q_{\mathbf{k}}(\omega) - \sum_{\zeta} \tilde{c}_{\mathbf{k},\zeta} \tilde{x}_{\mathbf{k},\zeta}(\omega) + \lambda_c \cos\phi_{\mathbf{k}} \omega_{\mathbf{k}} \mathcal{S}(\omega) = 0, \tag{S34b}$$

$$(-\omega^2 + \tilde{\omega}_{\mathbf{k},\zeta}^2) \tilde{x}_{\mathbf{k},\zeta}(\omega) - \tilde{c}_{\mathbf{k},\zeta} q_{\mathbf{k}}(\omega) = 0, \tag{S34c}$$

where V'_ω is the Fourier transform of $\partial V(\{R_j\})/\partial \mathcal{S}$. Plugging Eq. S34c into S34b to cancel the $\tilde{x}_{\mathbf{k},\zeta}(\omega)$ terms, one obtains

$$\left[\omega_{\mathbf{k}}^2 - \omega^2 \left(1 + \sum_{\zeta} \frac{\tilde{c}_{\mathbf{k},\zeta}^2}{\tilde{\omega}_{\mathbf{k},\zeta}^2 (-\omega^2 + \tilde{\omega}_{\mathbf{k},\zeta}^2)} \right) \right] q_{\mathbf{k}}(\omega) + \lambda_c \cos\phi_{\mathbf{k}} \omega_{\mathbf{k}} \mathcal{S}(\omega) = 0. \tag{S35}$$

We further define

$$L_{\mathbf{k}}(\omega) = -\omega^2 \left[1 + \sum_{\zeta} \frac{\tilde{c}_{\mathbf{k},\zeta}^2}{\tilde{\omega}_{\mathbf{k},\zeta}^2 (-\omega^2 + \tilde{\omega}_{\mathbf{k},\zeta}^2)} \right], \tag{S36}$$

and Eq. S35 becomes

$$q_{\mathbf{k}}(\omega) = -\frac{\lambda_c \cos\phi_{\mathbf{k}} \omega_{\mathbf{k}} \mathcal{S}(\omega)}{\omega_{\mathbf{k}}^2 + L_{\mathbf{k}}(\omega)}. \tag{S37}$$

Notice that $L_{\mathbf{k}}(\omega)$ can be alternatively expressed as

$$L_{\mathbf{k}}(\omega) = -\omega^2 \left[1 + \int_0^{+\infty} ds \frac{\sum_{\zeta} \frac{\tilde{c}_{\mathbf{k},\zeta}^2}{\tilde{\omega}_{\mathbf{k},\zeta}} \delta(s - \tilde{\omega}_{\mathbf{k},\zeta})}{s(s^2 - \omega^2)} \right] = -\omega^2 \left[1 + \frac{2}{\pi} \int_0^{+\infty} ds \frac{J_{\text{loss}}(s, \mathbf{k})}{s(s^2 - \omega^2)} \right], \tag{S38}$$

where we used Eq. S10 to give rise to the loss spectral density function. Plugging Eq. S37 into S34a, one obtains

$$K(\omega)\mathcal{S}(\omega) \equiv \left(-\omega^2 + \sum_{\mathbf{k}} \frac{\lambda_c^2 \cos^2\phi_{\mathbf{k}} L_{\mathbf{k}}(\omega)}{\omega_{\mathbf{k}}^2 + L_{\mathbf{k}}(\omega)} \right) \mathcal{S}(\omega) = -V'_\omega, \tag{S39}$$

And the total spectral density function felt by the reaction coordinate $\mathcal{S}$ is given by the branch cut of $K(z)$ on the complex plane, $J(\omega) = \lim_{\epsilon \to 0^+} \text{Im}[K(\omega - i\epsilon)]$. For our Eq. S39, it reads as [13, 15]

$$J_{\text{eff}}(\omega_{\mathbf{k}}, \omega) \equiv \frac{\pi}{2} \sum_{\zeta} \frac{\tilde{c}_{\mathbf{k},\zeta}^2}{\tilde{\omega}_{\mathbf{k},\zeta}} \delta(\omega - \tilde{\omega}_{\mathbf{k},\zeta}) = \frac{\lambda_c^2 \cos^2\phi_{\mathbf{k}} \omega_{\mathbf{k}}^2 J_{\text{loss}}(\omega, \mathbf{k})}{\left[\omega_{\mathbf{k}}^2 - \omega^2 + \xi_{\mathbf{k}}(\omega)\right]^2 + [J_{\text{loss}}(\omega, \mathbf{k})]^2}, \tag{S40}$$

where $\xi_{\mathbf{k}}(\omega)$ is expressed as

$$\xi_{\mathbf{k}}(\omega) = \frac{2\omega^2}{\pi}\mathcal{P}\int_0^\infty ds\ \frac{J_{\mathrm{loss}}(s,\mathbf{k})}{s(\omega^2 - s^2)}. \tag{S41}$$

And $\mathcal{P}$ in the above expression denotes the principal value integral. Notice that Eq. S40 is additive with respect to $\mathbf{k}$, meaning the total spectral density function is contributed by all of the cavity modes as well as their associated loss. Further, the normal mode transformations are restricted by the following identities [13]

$$\lambda_{\mathrm{c}}\cos\phi_{\mathbf{k}}\omega_{\mathbf{k}}\hat{q}_{\mathbf{k}} = \sum_\zeta \tilde{c}_{\mathbf{k},\zeta}\hat{\tilde{x}}_{\mathbf{k},\zeta}, \qquad \sum_\zeta \tilde{c}^2_{\mathbf{k},\zeta} = \lambda_{\mathrm{c}}^2\cos^2\phi_{\mathbf{k}}\omega^2_{\mathbf{k}}, \qquad \omega_{\mathbf{k}} = \lambda_{\mathrm{c}}^2\cos^2\phi_{\mathbf{k}}\omega^2_{\mathbf{k}}\cdot\left(\sum_\zeta \tilde{c}^2_{\mathbf{k},\zeta}/\tilde{\omega}^2_{\mathbf{k},\zeta}\right)^{-1}. \tag{S42}$$

To simplify our argument, we assume that the cavity loss is homogeneous (*i.e.*, does not depend on $\mathbf{k}$) and strictly Ohmic (*i.e.*, Markovian), which means

$$J_{\mathrm{loss}}(\omega,\mathbf{k}) = \alpha\omega\exp(-\omega/\omega_{\mathrm{m}}), \quad \omega_{\mathrm{m}} \to +\infty, \tag{S43}$$

where $\alpha \equiv \tau_{\mathrm{c}}^{-1}$ is the inverse of the cavity lifetime τ_{c}. Under the Markovian limit, $\xi_{\mathbf{k}}(\omega) \to 0$, Eq. S40 is simplified as

$$J_{\mathrm{eff}}(\omega_{\mathbf{k}},\omega) = \frac{\lambda_{\mathrm{c}}^2\cos^2\phi_{\mathbf{k}}\omega^2_{\mathbf{k}}\tau_{\mathrm{c}}^{-1}\omega}{\left(\omega^2_{\mathbf{k}} - \omega^2\right)^2 + \tau_{\mathrm{c}}^{-2}\omega^2}, \tag{S44}$$

which is Eq. 12 of the main text.

V. DERIVATION OF THE FGR RATE UNDER MANY-MODES CASES

We begin by deriving the FGR rate constant expression for a single molecule coupled to a specific cavity mode $\omega_{\mathbf{k}}$. The same derivation can be found in Appendix A of Ref. 16. Using FGR, the cavity promoted transition from $|\nu_{\mathrm{L}}\rangle \to |\nu'_{\mathrm{L}}\rangle$ is

$$\begin{aligned}\tilde{k}_{\mathrm{VSC}}(\omega_{\mathbf{k}},\omega_0) &= 2\pi\sum_\zeta\sum_{n_\zeta}|\langle\nu_{\mathrm{L}}, n_\zeta+1|\mu_{\mathrm{LL'}}\hat{\sigma}_x\otimes\left(\tilde{c}_{\mathbf{k},\zeta}\hat{\tilde{x}}_{\mathbf{k},\zeta}\right)|\nu'_{\mathrm{L}}, n_\zeta\rangle|^2\cdot\frac{e^{-(n_\zeta+1)\beta\tilde{\omega}_{\mathbf{k},\zeta}}}{\mathcal{Z}_\zeta}\delta(\omega_0 - \tilde{\omega}_{\mathbf{k},\zeta}) \\ &= 2\pi\mu^2_{\mathrm{LL'}}\sum_\zeta\frac{\tilde{c}^2_{\mathbf{k},\zeta}}{2\tilde{\omega}_{\mathbf{k},\zeta}}\delta(\omega_0 - \tilde{\omega}_{\mathbf{k},\zeta})\sum_{n_\zeta}\frac{e^{-(n_\zeta+1)\beta\tilde{\omega}_{\mathbf{k},\zeta}}}{\mathcal{Z}_\zeta}(n_\zeta+1) \\ &= 2\mu^2_{\mathrm{LL'}}\cdot J_{\mathrm{eff}}(\omega_{\mathbf{k}},\omega_0)\cdot n(\omega_0),\end{aligned} \tag{S45}$$

where $|n_\zeta\rangle$ denote the n_{th} Fock state of the effective normal mode $\hat{\tilde{x}}_{\mathbf{k},\zeta}$, and $\mathcal{Z}_\zeta = \sum_{n_\zeta} e^{-n_\zeta\beta\tilde{\omega}_{\mathbf{k},\zeta}}$ is the partition function for the thermal distribution of mode $\hat{\tilde{x}}_{\mathbf{k},\zeta}$. In the last line of Eq. S45 we have defined the Bose-Einstein distribution function as below,

$$n(\omega) = \sum_{n_\zeta}\frac{e^{-(n_\zeta+1)\beta\omega}}{\mathcal{Z}_\zeta}(n_\zeta+1) = \frac{1}{e^{\beta\omega}-1}. \tag{S46}$$

In the last line of Eq. S45, the highly peaked delta function makes $\tilde{\omega}_{\mathbf{k},\zeta}$ to be ω_0, and $n(\tilde{\omega}_{\mathbf{k},\zeta}) \to n(\omega_0)$, and we have used the definition of the effective spectral density function (Eq. S40) in the discrete form.

With many cavity modes inside a thermalized cavity, we further consider the thermal statistics among the different cavity photon modes with frequency $\omega_{\mathbf{k}}$,

$$\mathcal{P}_{\mathbf{k}} = e^{-\beta\hbar\omega_{\mathbf{k}}}/\mathcal{Z}. \tag{S47}$$

For each mode $\omega_{\mathbf{k}}$, the FGR rate is $\tilde{k}_{\mathrm{VSC}}(\omega_{\mathbf{k}},\omega_0)$. The overall FGR rate with single molecule coupling to many cavity modes is a weighted average of the single mode rates, which is evaluated via

$$k_{\mathrm{VSC}}(\omega_0) = \sum_{\mathbf{k}}\mathcal{P}_{\mathbf{k}}\cdot\tilde{k}_{\mathrm{VSC}}(\omega_{\mathbf{k}},\omega_0) = 2\mu^2_{\mathrm{LL'}}\sum_{\mathbf{k}}\mathcal{P}_{\mathbf{k}}\cdot J_{\mathrm{eff}}(\omega_{\mathbf{k}},\omega_0)\cdot n(\omega_0). \tag{S48}$$

For many-molecules cases, the above $k_{\rm VSC}(\omega_0)$ need to be generalized to

$$k_{\rm VSC} = \frac{1}{N}\frac{2}{\hbar}\sum_{j=1}^{N}\left|\langle\nu_j|\hat{\mathcal{S}}|G\rangle\right|^2 \cdot \sum_{\mathbf{k}}\mathcal{P}_{\mathbf{k}} \cdot J_{\rm eff}(\omega_{\mathbf{k}},\omega_0)\cdot n(\omega_0), \tag{S49}$$

which is just Eq. 13 of the main text.

VI. THE DENSITY OF STATES AND THE EFFECTIVE SPECTRAL DENSITY

The cavity dispersion relation is written as (cf. Eq. 2 of the main text)

$$\omega_{\mathbf{k}} = \frac{c}{n_{\rm c}}\sqrt{k_\perp^2 + k_\parallel^2} = \frac{ck_\perp}{n_{\rm c}}\sqrt{1+\tan^2\theta}, \tag{S50}$$

where $\tan\theta = k_\parallel/k_\perp$. In turn, one can calculate $k_\parallel$ in terms of the cavity frequency ω as

$$k_\parallel = \pm\frac{n_{\rm c}\sqrt{\omega_{\mathbf{k}}^2-\omega_{\rm c}^2}}{c}, \tag{S51}$$

where for $k_\parallel = 0$ we introduce

$$\omega_{\rm c} = ck_\perp/n_{\rm c}, \tag{S52}$$

which is the photon frequency associated with the quantized direction (normal incidence). Evaluating the derivative of $k_\parallel$ with respect to $\omega_{\mathbf{k}}$, one obtains

$$\frac{dk_\parallel}{d\omega_{\mathbf{k}}} = \pm\frac{n_{\rm c}\omega_{\mathbf{k}}}{c\sqrt{\omega_{\mathbf{k}}^2-\omega_{\rm c}^2}}. \tag{S53}$$

For a volume $V_{\rm D} = L^{\rm D}$, periodic boundary conditions give the allowed wavevectors as $k_{n_j} = \frac{2\pi}{L}n_j,\ n_j \in \mathbb{Z},\ j = 1,\cdots,{\rm D}$, where D is the dimension, with a uniform spacing as $\Delta k_\parallel = \frac{2\pi}{L}$. We define the photonic density of states (DOS) $g_{\rm D}(\omega)$ for a D-dimensional FP cavity as

$$g_{\rm D}(\omega) = \int_{-\infty}^{+\infty}\frac{dk^{\rm D}}{(\Delta k_\parallel)^{\rm D}}\ \delta(\omega-\omega_{\mathbf{k}}), \tag{S54}$$

such that $g_{\rm D}(\omega)d\omega$ is the number of modes with frequencies between ω and $\omega + d\omega$. Eq. S54 can be evaluated using Eqs. S51 and S53. For example, the 1D DOS reads as

$$\begin{aligned} g_{\rm 1D}(\omega) &= \frac{1}{\Delta k_\parallel}\int_{-\infty}^{+\infty} dk\ \delta(\omega-\omega_{\mathbf{k}}) \\ &= \frac{2}{\Delta k_\parallel}\int_0^{\infty} d\omega_{\mathbf{k}}\ \frac{dk_\parallel}{d\omega_{\mathbf{k}}}\ \delta(\omega-\omega_{\mathbf{k}}) \\ &= \frac{2}{\Delta k_\parallel}\frac{n_{\rm c}}{c}\frac{\omega}{\sqrt{\omega^2-\omega_{\rm c}^2}}\cdot\Theta(\omega-\omega_{\rm c}), \end{aligned} \tag{S55}$$

where $\Theta(\omega-\omega_{\rm c})$ is the Heaviside function, which takes the value $\Theta(\omega-\omega_{\rm c}) = 1$ when $\omega \geq \omega_{\rm c}$ and $\Theta(\omega-\omega_{\rm c}) = 0$ when $\omega < \omega_{\rm c}$, such that there is no mode density below $\omega_{\rm c}$. Eq. S55 is just Eq. 24 of the main text if one takes $n_{\rm c} = 1$. Similarly, the 2D DOS reads as

$$\begin{aligned} g_{\rm 2D}(\omega) &= \frac{1}{(\Delta k_\parallel)^2}\int dk^2\ \delta(\omega-\omega_{\mathbf{k}}) \\ &= \frac{1}{(\Delta k_\parallel)^2}\int_0^{2\pi} d\phi_{\mathbf{k}}\int_0^{\infty} dk_\parallel\ k_\parallel\delta(\omega-\omega_{\mathbf{k}}) \\ &= \frac{2\pi}{(\Delta k_\parallel)^2}\int_0^{\infty} d\omega_{\mathbf{k}}\ \left|\frac{dk_\parallel}{d\omega_{\mathbf{k}}}\right|\frac{n_{\rm c}\sqrt{\omega_{\mathbf{k}}^2-\omega_{\rm c}^2}}{c}\delta(\omega-\omega_{\mathbf{k}}) \\ &= \frac{2\pi}{(\Delta k_\parallel)^2}\int_0^{\infty} d\omega_{\mathbf{k}}\ \frac{n_{\rm c}\omega_{\mathbf{k}}}{c\sqrt{\omega_{\mathbf{k}}^2-\omega_{\rm c}^2}}\cdot\frac{n_{\rm c}\sqrt{\omega_{\mathbf{k}}^2-\omega_{\rm c}^2}}{c}\delta(\omega-\omega_{\mathbf{k}}) \\ &= \frac{2\pi}{(\Delta k_\parallel)^2}\left(\frac{n_{\rm c}}{c}\right)^2\omega\cdot\Theta(\omega-\omega_{\rm c}), \end{aligned} \tag{S56}$$

being Eq. 27 of the main text if one replaces n_c with 1. More generally, one can prove that

$$g_D(\omega) = \frac{1}{(\Delta k_\parallel)^D}\frac{D\pi^{D/2}}{(D/2)!}\left(\frac{n_c}{c}\right)^D(\omega^2-\omega_c^2)^{\frac{D-2}{2}}\omega\cdot\Theta(\omega-\omega_c), \tag{S57}$$

where $x!$ means the Gamma function $\Gamma(x+1)$ and $(1/2)! = \Gamma(3/2) = \sqrt{\pi}/2$.

For a quasi-continuous wave vector $\mathbf{k}$ inside a FP cavity, we can rewrite the summation in Eq. S49 into integration,

$$\sum_{\mathbf{k}} f(\mathbf{k}) \to \int d\omega\ g_D(\omega)f(\omega). \tag{S58}$$

For 1D cavity, we have $\cos\phi_{\mathbf{k}} = 1$, so that

$$\begin{aligned} k_{\mathrm{VSC}} \propto \mathcal{A}(\omega_0) &= \frac{1}{\mathcal{Z}}\sum_{\mathbf{k}}\frac{\omega_{\mathbf{k}}^2\tau_c^{-1}\omega_0}{(\omega_{\mathbf{k}}^2-\omega_0^2)^2+\tau_c^{-2}\omega_0^2}\cdot e^{-\beta\hbar\omega_{\mathbf{k}}} \\ &= \frac{1}{\mathcal{Z}}\int d\omega\ g_{1D}(\omega)\frac{\omega^2\tau_c^{-1}\omega_0}{(\omega^2-\omega_0^2)^2+\tau_c^{-2}\omega_0^2}\cdot e^{-\beta\hbar\omega} = \frac{\int_{\omega_c}^{\omega_m} d\omega\ \frac{\omega}{\sqrt{\omega^2-\omega_c^2}}\frac{\omega^2\tau_c^{-1}\omega_0}{(\omega^2-\omega_0^2)^2+\tau_c^{-2}\omega_0^2}e^{-\beta\hbar\omega}}{\int_{\omega_c}^{\omega_m} d\omega\ \frac{\omega}{\sqrt{\omega^2-\omega_c^2}}e^{-\beta\hbar\omega}}, \end{aligned} \tag{S59}$$

where ω_m is the upper limit of integration, being Eq. 25 of the main text. In Eq. S59, the integral is dominated by $\omega = \omega_c$ since there is a singularity in the factor of $\frac{\omega}{\sqrt{\omega^2-\omega_c^2}}$, which can be approximated as

$$\frac{1}{\omega_m}\frac{\omega}{\sqrt{\omega^2-\omega_c^2}} \approx \delta(\omega-\omega_c) \tag{S60}$$

when the cutoff frequency $\omega_m \to \infty$. As a result, with the approximation made in Eq. S60, one can obtain from Eq. S59 that

$$\mathcal{A}(\omega_0) = \frac{\omega_c^2\tau_c^{-1}\omega_0}{(\omega_c^2-\omega_0^2)^2+\tau_c^{-2}\omega_0^2}, \tag{S61}$$

which is Eq. 26 of the main text. Note that Eq. S61 reduces to the single mode case. We also present numerical integration results versus the analytic results to verify this approximation is valid. Fig. S2a presents the FGR rates under various light-matter coupling strength obtained using the analytical expression in Eq. 35 of the main text, corresponding to the single mode case where $\mathcal{A}(\omega_0)$ is given in Eq. S61. And Fig. S2b corresponds to the results using numerical integration in Eq. S59. The cavity lifetime is fixed at $\tau_c = 50$ fs. The FGR results are all rescaled by a factor of 0.4 to keep in line with the main text. In Fig. S2b, the numerical integration scheme is the same as those mentioned in the main text. One can see that they are almost identical to each other within the light-matter coupling strengths we presented. As a result, the approximation in Eq. 26 of the main text is justified.

The validation of single-mode approximation in the VSC problem will greatly benefit us to simplify the analysis. Physically, it means that the DOS at $\theta \neq 0$ is infinitesimal compared to the DOS at $\theta = 0$. But this is not contrary to the fact that there is still Rabi-splitting when $\omega_0 > \omega_c$ because there are indeed modes (and associated photonic states) at that higher frequency.

Nevertheless, when directly generalizing the above analysis for a 2D FP cavity (and considering only one polarization direction), the theory will not be satisfying to explain the normal incidence condition. This is because that the 2D DOS $g_{2D}(\omega)$ (Eq. S56) does not have any singularity, so that the result is no longer dominated by the $k_\parallel = 0$ contribution. This problem can be fixed by introducing additional mode lifetime corrections as is discussed in Sec. 3.2 of the main text.

We further provide the proof for $g'_{2D}(\omega) = g_{2D}(\omega)/2$, see Eqs. 20-21 of the main text.

$$\begin{aligned} g'_{2D}(\omega) &= \frac{1}{(\Delta k_\parallel)^2}\int dk^2\ \delta(\omega-\omega_{\mathbf{k}})\cdot\cos^2\phi_{\mathbf{k}} \\ &= \frac{1}{(\Delta k_\parallel)^2}\int_0^\infty dk\ k\delta(\omega-\omega_{\mathbf{k}})\cdot\int_0^{2\pi} d\phi_{\mathbf{k}}\ \cos^2\phi_{\mathbf{k}} \\ &= \frac{1}{(\Delta k_\parallel)^2}\int_0^\infty dk\ k\delta(\omega-\omega_{\mathbf{k}})\cdot\frac{1}{2}\int_0^{2\pi} d\phi_{\mathbf{k}} \\ &= \frac{1}{2}\frac{1}{(\Delta k_\parallel)^2}\int dk^2\ \delta(\omega-\omega_{\mathbf{k}}) = \frac{1}{2}g_{2D}(\omega). \end{aligned} \tag{S62}$$

Since $g'_{2D}(\omega)$ and $g_{2D}(\omega)$ only differ by a constant 1/2 factor, we regard them interchangable.

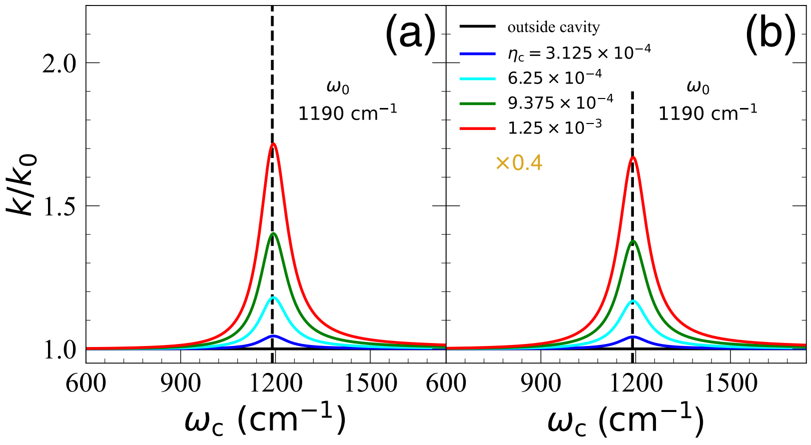

FIG. S2. FGR rates under various light-matter coupling strength (all rescaled by a factor of 0.4). The cavity lifetime is fixed at $\tau_c = 50$ fs. (a) Results using the analytical expression in Eq. 35 of the main text. (b) Results using numerical integration in Eq. S59.

VII. QUANTUM DYNAMICS SIMULATIONS OF THE LOSSY CAVITY, AND THE RATE CONSTANT

In Fig. 5 of the main text, we use the numerically exact hierarchical equations of motion (HEOM) approach [17–20] to propagate the quantum dynamics of the VSC model described in Sec. II. The single molecule, single mode Hamiltonian is expressed as

$$\hat{H} = \frac{\hat{P}^2}{2M} + V(\hat{R}) + \hat{H}_\nu + \hat{H}_{\rm eff}, \tag{S63}$$

where $\hat{H}_\nu$ is the dissipative system-bath Hamiltonian that describes the linear coupling between reaction coordinate $\hat{R}$ and phonon bath, expressed as follows

$$\hat{H}_\nu = \frac{1}{2}\sum_i \left[\hat{p}_i^2 + \omega_i^2\left(\hat{x}_i - \frac{c_i}{\omega_i^2}\hat{R}\right)^2\right], \tag{S64}$$

and described by the Drude-Lorentz spectral density function,

$$J_\nu(\omega) \equiv \frac{\pi}{2}\sum_j \frac{c_j^2}{\omega_j}\delta(\omega - \omega_j) = \frac{2\lambda_\nu\gamma_\nu\omega}{\omega^2 + \gamma_\nu^2}. \tag{S65}$$

We follow Ref. 10 by taking $\gamma_\nu = 200$ cm^{-1} and $\lambda_\nu = 0.1\omega_{\rm b}\gamma_\nu/2$. The cavity and its associated loss are combined as

$$\hat{H}_{\rm eff} = \frac{1}{2}\sum_j \left[\hat{\tilde{P}}_j^2 + \tilde{\Omega}_j^2\left(\hat{\tilde{X}}_j - \frac{\tilde{C}_j}{\tilde{\Omega}_j^2}\hat{R}\right)^2\right], \tag{S66}$$

with the effective spectral density function [13, 15] expressed as belows

$$J_{\rm eff}(\omega) \equiv \frac{\pi}{2}\sum_j \frac{\tilde{C}_j^2}{\tilde{\Omega}_j}\delta(\omega - \tilde{\Omega}_j) = \frac{2\alpha\eta_c^2\omega_c^3\omega}{(\omega_c^2 - \omega^2)^2 + \alpha^2\omega^2}, \tag{S67}$$

where $\tau_c = 1/\alpha$ is the cavity lifetime, which is fixed as 200 fs in our simulation. η_c is the light-matter coupling strength that defined in Eq. 38 of the main text, which varies from 3.125×10^{-4} to 1.25×10^{-3}, a similar range of parameters as used in Ref. 10. In Eq. S40, we explicitly take the Markovian limit for the photon-loss bath, so that the effective spectral density is in the form of a Brownian oscillator. For a practical calculation, truncation has to be made upon the number of matter states, restricting the dynamics in a relatively low-energy subspace while ensuring numerical accuracy. Here, we use the lowest $\mathcal{F} = 10$ vibrational eigenstates to construct the matter Hilbert subspace (see Sec. II.)

For the HEOM propagation, there are several convergence control parameters, including (1) the number of bath terms obtained from the decomposition of the bare environment time-correlation function (TCF), (2) the time step for integration, (3) the depth of the EOMs (or the number of tiers), and (4) on-the-fly filtering [21] error tolerance. We have carefully checked all of the above convergence parameters. More specifically, we use the fourth order Runge-Kutta (RK-4) integrator with a time step of 0.025 fs, together with the on-the-fly filtering algorithm [21] with an error tolerance of 1×10^{-7}.

The HEOM method requires a factorizable initial condition between the system and bath subspaces. Note that the choice of a particular initial condition will not influence the rate dynamics or rate constant. [8, 22] We thus assume a factorizable initial full-density matrix as

$$\hat{\rho}(0) = |\nu_{\mathrm{L}}\rangle\langle\nu_{\mathrm{L}}| \otimes \frac{e^{-\beta \hat{h}_{\mathrm{B}}^{\mathrm{eff}}}}{Z_{\mathrm{B}}}, \tag{S68}$$

where $Z_{\mathrm{B}} \equiv \mathrm{Tr}_{\mathrm{B}}[e^{-\beta \hat{h}_{\mathrm{B}}^{\mathrm{eff}}}]$ is the bath partition function, $\mathrm{Tr}_{\mathrm{B}}[\cdot]$ denotes taking the partial trace over the bath DOFs, and $\hat{h}_{\mathrm{B}}^{\mathrm{eff}} = \frac{1}{2}\sum_i \left(\hat{p}_i^2 + \omega_i^2 \hat{x}_i^2\right) + \frac{1}{2}\sum_j \left(\hat{\tilde{P}}_j^2 + \tilde{\Omega}_j^2 \hat{\tilde{X}}_j^2\right)$ is the bare effective bath Hamiltonian (c.f. Eq. S29). We adopt the initial condition of Eq. S68 in all of our numerical simulations with HEOM. The reduced density matrix of the system, on the other hand, is defined as

$$\hat{\rho}_{\mathrm{S}}(t) = \mathrm{Tr}_{\mathrm{B}}\left[\hat{\rho}(t)\right], \tag{S69}$$

where $\hat{\rho}(t)$ is the full density matrix at time t.

In order to evaluate the forward rate constant, we follow the previous work [8, 10, 23] by defining the time-dependent reactant ($\mathcal{R}$) and product state ($\mathcal{P}$) populations as

$$P_{\mathcal{R}}(t) = \mathrm{Tr}_{\mathrm{S}}\left[(1-\hat{h})\hat{\rho}_{\mathrm{S}}(t)\right], \tag{S70a}$$

$$P_{\mathcal{P}}(t) = 1 - P_{\mathcal{R}}(t), \tag{S70b}$$

where the trace Tr_{S} in Eq. S70a is performed along the system DOF (which is the reaction coordinate R for the model considered here). In Eq. S70, $\hat{h} = h(\hat{R} - R^{\ddagger})$ is the Heaviside operator that projects onto the product states, where $h(R) = 1$ for $R > R^{\ddagger}$ (in the product region) and $h(R) = 0$ for $R < R^{\ddagger}$ (in the reaction region), $R^{\ddagger}$ is the dividing surface. For the symmetric DW model considered here, we use $R^{\ddagger} = 0$. Under the system's eigen-representation $\{|\nu_i\rangle\}$ in the truncated $\mathcal{F}$-dimensional Hilbert subspace, it is evaluated as

$$P_{\mathcal{R}}(t) = \sum_{j=1}^{\mathcal{F}} \langle\nu_j|(1-\hat{h})\hat{\rho}_{\mathrm{S}}(t)|\nu_j\rangle = \sum_{i,j=1}^{\mathcal{F}} \langle\nu_j|(1-\hat{h})|\nu_i\rangle \cdot [\hat{\rho}_{\mathrm{S}}]_{ij}(t),$$

where $[\hat{\rho}_{\mathrm{S}}]_{ij}(t) = \langle\nu_i|\hat{\rho}_{\mathrm{S}}(t)|\nu_j\rangle$ is the system reduced density matrix element, and $\langle\nu_j|(1-\hat{h})|\nu_i\rangle$ is evaluated as

$$\begin{aligned}\langle\nu_j|(1-\hat{h})|\nu_i\rangle &= \int_{-\infty}^{+\infty} dR\ \psi_j^*(R)[1 - h(R - R^{\ddagger})]\psi_i(R) \\ &= \int_{-\infty}^{R^{\ddagger}} dR\ \psi_j^*(R)\psi_i(R),\end{aligned}$$

where $\psi_i(R) = \langle R|\nu_i\rangle$, $\psi_j^*(R) = \langle\nu_j|R\rangle$ are the eigenfunctions of the vibrational eigenstates in the position representation (obtained using DVR grid-based method). The forward rate constant is then evaluated via [8, 15, 23, 24]

$$k = -\lim_{t\to t_{\mathrm{p}}} \frac{\dot{P}_{\mathcal{R}}(t)}{P_{\mathcal{R}}(t) + \chi_{\mathrm{eq}} \cdot [P_{\mathcal{R}}(t) - 1]}, \tag{S71}$$

where $\chi_{\mathrm{eq}} \equiv \langle P_{\mathcal{R}}\rangle / \langle P_{\mathcal{P}}\rangle$ denotes the ratio of equilibrium population between the reactant and product. The time derivative $\dot{P}_{\mathcal{R}}(t)$ is evaluated numerically.

On the other hand, k_{VSC} is evaluated via the analytic FGR expression in Eq. 35 of the main text, where we adopted a convolution between the original FGR expression and an inhomogeneous broadening function which is assumed to have a Gaussian profile whose variance is expressed as [25]

$$\sigma^2 = \epsilon_z^2 \cdot \frac{1}{\pi} \int_0^{\infty} d\omega\ J_{\nu}(\omega) \coth(\beta\omega/2), \tag{S72}$$

where $\epsilon_z = \langle \nu'_\mathrm{L}|\hat{R}|\nu'_\mathrm{L}\rangle - \langle \nu_\mathrm{L}|\hat{R}|\nu_\mathrm{L}\rangle$. This broadening is due to the molecular phonon bath $J_\nu(\omega)$. Unfortunately, the integral in Eq. S72 is divergent for the Drude-Lorentz spectral density given in Eq. S65, so we instead take the upper limit of the integral to be the characteristic frequency $\gamma_\nu = 200$ cm^{-1} of the dissipative molecular phonon bath $J_\nu(\omega)$, which is in line with the *low-frequency modes* that brings about the inhomogeneous broadening. For the model parameters we used, the variance is evaluated as $\sigma = 30.74$ cm^{-1} via numerical integration.

VIII. EFFECTS OF THE $\mathcal{D}/c$ VALUES ON THE RATE PROFILES FOR THE 2D CAVITY CASE

We further show the effects of $\mathcal{D}/c$ values to the rate profile k/k_0 inside a 2D FP cavity. Fig. S3 presents the VSC rate profiles k/k_0 for the 2D cavity case in panels (b)-(d), among which panel (c) is the same as Fig. 6b of the main text, while panel (a) is the same as Fig. 6a of the main text. All of the VSC rates are uniformly rescaled with a factor of 0.4 to keep in line with the main text. Fig. S3b-d shows the numerical behavior of the rate profiles under different η_c and $\mathcal{D}/c$ values, among which $\mathcal{D}/c = 1$ fs, 3.33 fs, and 33.3 fs, corresponding to $\mathcal{D} = 300$ nm, 1 μm, and 10 μm, respectively. One can see that the maximal contribution is still around $k_\parallel = 0$, although no singularity is present. The width of the rate profiles become wider, the peak become lower, and the peak position gradually move away from $\omega_\mathrm{c} = \omega_0$ as $\mathcal{D}/c$ increases, causing red-shifts and is unsatisfying to explain the resonance condition at the normal incidence. This is because when $\mathcal{D}/c$ increases, the weighting factor $F(\omega)$ (see Eq. 32 of the main text) is less sensitive to the incident angle θ, and Γ'_{10} is less dominant.

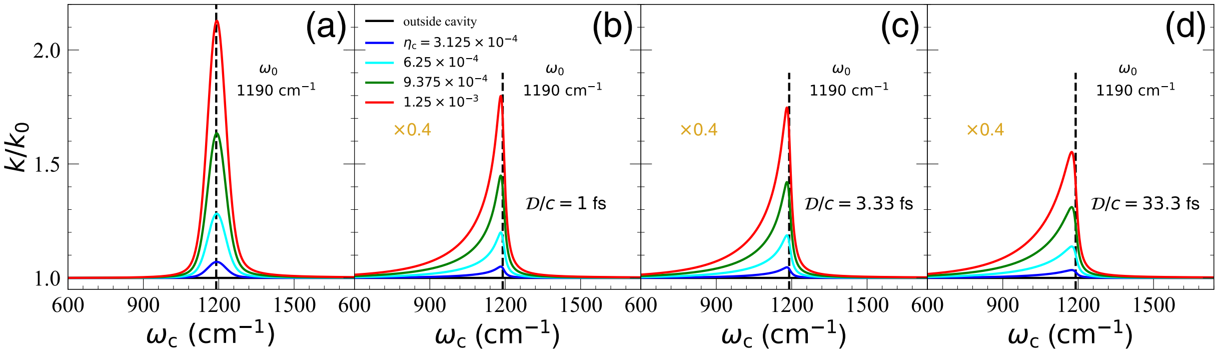

FIG. S3. (a) VSC rate profiles k/k_0 under different η_c values for the 1D case, which is the same as Fig. 6a of the main text. (b)-(d) VSC rate profiles k/k_0 under different η_c and $\mathcal{D}/c$ values for the 2D cavity case, among which $\mathcal{D}/c = 1$ fs, 3.33 fs, and 33.3 fs, corresponding to $\mathcal{D} = 300$ nm, 1 μm, and 10 μm, respectively. Here we fix $\tau_\mathrm{c} = 200$ fs. All of the VSC rates are rescaled with a factor of 0.4 to keep in consistence with the main text.

[1] E. A. Power and S. Zienau, Coulomb gauge in non-relativistic quantum electro-dynamics and the shape of spectral lines, Philos. Trans. Royal Soc. A **251**, 427 (1959).
[2] C. Cohen-Tannoudji, J. Dupont-Roc, and G. Grynberg, *Photons and Atoms: Introduction to Quantum Electrodynamics* (Wiley, 1997).
[3] R. G. Woolley, A reformulation of molecular quantum electrodynamics, J. Phys. B: At. Mol. Phys. **7**, 488 (1974).
[4] A. Mandal, M. Taylor, B. Weight, E. Koessler, X. Li, and P. Huo, Theoretical advances in polariton chemistry and molecular cavity quantum electrodynamics, ChemRxiv , 10.26434/chemrxiv (2022).
[5] J. Keeling, *Light-Matter Interactions and Quantum Optics* (University of St. Andrews, 2012).
[6] A. Caldeira and A. Leggett, Quantum tunnelling in a dissipative system, Ann. Physics **149**, 374 (1983).
[7] M. Topaler and N. Makri, Quantum rates for a double well coupled to a dissipative bath: Accurate path integral results and comparison with approximate theories, J. Chem. Phys. **101**, 7500 (1994).
[8] Q. Shi, L. Zhu, and L. Chen, Quantum rate dynamics for proton transfer reaction in a model system: Effect of the rate promoting vibrational mode, J. Chem. Phys. **135**, 044505 (2011).
[9] D. T. Colbert and W. H. Miller, A novel discrete variable representation for quantum mechanical reactive scattering via the s-matrix kohn method, J. Chem. Phys. **96**, 1982 (1992).
[10] L. P. Lindoy, A. Mandal, and D. R. Reichman, Quantum dynamical effects of vibrational strong coupling in chemical reactivity, Nat. Commun. **14**, 2733 (2023).

[11] A. Thomas, A. Jayachandran, L. Lethuillier-Karl, R. M. Vergauwe, K. Nagarajan, E. Devaux, C. Genet, J. Moran, and T. W. Ebbesen, Ground state chemistry under vibrational strong coupling: dependence of thermodynamic parameters on the rabi splitting energy, Nanophotonics **9**, 249 (2020).
[12] A. Garg, J. N. Onuchic, and V. Ambegaokar, Effect of friction on electron transfer in biomolecules, J. Chem. Phys. **83**, 4491 (1985).
[13] M. Thoss, H. Wang, and W. H. Miller, Self-consistent hybrid approach for complex systems: Application to the spin-boson model with debye spectral density, J. Chem. Phys. **115**, 2991 (2001).
[14] A. J. Leggett, Quantum tunneling in the presence of an arbitrary linear dissipation mechanism, Phys. Rev. B **30**, 1208 (1984).
[15] J. E. Lawrence, T. Fletcher, L. P. Lindoy, and D. E. Manolopoulos, On the calculation of quantum mechanical electron transfer rates, J. Chem. Phys. **151**, 114119 (2019).
[16] W. Ying and P. Huo, Resonance theory and quantum dynamics simulations of vibrational polariton chemistry, ChemRxiv , 10.26434/chemrxiv (2023).
[17] Y. Tanimura, Nonperturbative expansion method for a quantum system coupled to a harmonic-oscillator bath, Phys. Rev. A **41**, 6676 (1990).
[18] Y. Tanimura, Stochastic liouville, langevin, fokker–planck, and master equation approaches to quantum dissipative systems, J. Phys. Soc. Jpn. **75**, 082001 (2006).
[19] R.-X. Xu, P. Cui, X.-Q. Li, Y. Mo, and Y. Yan, Exact quantum master equation via the calculus on path integrals, J. Chem. Phys. **122**, 041103 (2005).
[20] R.-X. Xu and Y. Yan, Dynamics of quantum dissipation systems interacting with bosonic canonical bath: Hierarchical equations of motion approach, Phys. Rev. E **75**, 031107 (2007).
[21] Q. Shi, L. Chen, G. Nan, R.-X. Xu, and Y. Yan, Efficient hierarchical liouville space propagator to quantum dissipative dynamics, J. Chem. Phys. **130**, 084105 (2009).
[22] A. Bose and N. Makri, Non-equilibrium reactive flux: A unified framework for slow and fast reaction kinetics, J. Chem. Phys. **147**, 152723 (2017).
[23] H. Wang, D. E. Skinner, and M. Thoss, Calculation of reactive flux correlation functions for systems in a condensed phase environment: A multilayer multiconfiguration time-dependent hartree approach, J. Chem. Phys. **125**, 174502 (2006).
[24] I. R. Craig, M. Thoss, and H. Wang, Proton transfer reactions in model condensed-phase environments: Accurate quantum dynamics using the multilayer multiconfiguration time-dependent hartree approach, J. Chem. Phys. **127**, 144503 (2007).
[25] P. Huo, I. Miller, Thomas F., and D. F. Coker, Communication: Predictive partial linearized path integral simulation of condensed phase electron transfer dynamics, J. Chem. Phys. **139**, 151103 (2013).
